# Field transcriptome revealed critical developmental and physiological transitions involved in the expression of growth potential in *japonica *rice

**DOI:** 10.1186/1471-2229-11-10

**Published:** 2011-01-12

**Authors:** Yutaka Sato, Baltazar Antonio, Nobukazu Namiki, Ritsuko Motoyama, Kazuhiko Sugimoto, Hinako Takehisa, Hiroshi Minami, Kaori Kamatsuki, Makoto Kusaba, Hirohiko Hirochika, Yoshiaki Nagamura

**Affiliations:** 1 National Institute of Agrobiological Sciences, Kannondai 2-1-2, Tsukuba, Ibaraki 305-8602, Japan; 2 Mitsubishi Space Software Co. Ltd., Takezono 1-6-1, Tsukuba, Ibaraki 305-0032, Japan; 3 Graduate School of Science, Hiroshima University, Higashi-Hiroshima, Hiroshima 739-8526, Japan

## Abstract

**Background:**

Plant growth depends on synergistic interactions between internal and external signals, and yield potential of crops is a manifestation of how these complex factors interact, particularly at critical stages of development. As an initial step towards developing a systems-level understanding of the biological processes underlying the expression of overall agronomic potential in cereal crops, a high-resolution transcriptome analysis of rice was conducted throughout life cycle of rice grown under natural field conditions.

**Results:**

A wide range of gene expression profiles based on 48 organs and tissues at various developmental stages identified 731 organ/tissue specific genes as well as 215 growth stage-specific expressed genes universally in leaf blade, leaf sheath, and root. Continuous transcriptome profiling of leaf from transplanting until harvesting further elucidated the growth-stage specificity of gene expression and uncovered two major drastic changes in the leaf transcriptional program. The first major change occurred before the panicle differentiation, accompanied by the expression of *RFT1*, a putative florigen gene in long day conditions, and the downregulation of the precursors of two microRNAs. This transcriptome change was also associated with physiological alterations including phosphate-homeostasis state as evident from the behavior of several key regulators such as miR399. The second major transcriptome change occurred just after flowering, and based on analysis of sterile mutant lines, we further revealed that the formation of strong sink, i.e., a developing grain, is not the major cause but is rather a promoter of this change.

**Conclusions:**

Our study provides not only the genetic basis for functional genomics in rice but also new insight into understanding the critical physiological processes involved in flowering and seed development, that could lead to novel strategies for optimizing crop productivity.

## Background

The high quality sequence of *Oryza sativa *L. ssp. *japonica *cv. Nipponbare genome elucidated the entire genetic blueprint of a major cereal crop that provides food for almost half the world population [1]. Subsequently, complete annotation of every trancriptional unit has become an enormous challenge not only for a complete understanding of the biology of rice, but more importantly, for efficient utilization of that information for genomics-based crop improvement [2-4]. Gene expression profiling is an important strategy for obtaining knowledge on presumed function of genes that comprise an organism [5]. Microarray analyses of the rice transcriptome encompassing different cell types [6], tissues and organs [7], specific stages of growth and development [8,9], and specific treatment conditions [10,11] have generated a large amount of information that provides initial clues for understanding the function of genes based on their time, place and level of expression in the plant.

Although rapid progress has been made during the last decade in understanding genes involved in developmental transitions, particularly the vegetative transition (juvenile-to-adult) and the transition to flowering [12-14], the physiological changes associated with phase transition have been poorly defined. Recently, microarray analysis of the vegetative transition in maize revealed that photosynthesis related genes are upregulated in the juvenile phase [15]. The changes in physiological state of the plant triggered by internal or external stimuli under natural field condition are thought to be reflected as corresponding changes in the transcriptome. However, the global configuration and complexity of the transcriptome that underlies physiological processes has not been scrutinized in sufficient depth particularly in a cereal crop. In order to understand these transcriptional programs reflecting physiological states it is essential to monitor the expression profiles of a large number of genes, including uncharacterized ones, throughout the life cycle of the rice plant in the field and to do this at high resolution.

Here, we establish a field transcriptome profile of the model rice cultivar, Nipponbare, by spatiotemporal gene expression analysis of 48 tissues and organs at various stages of growth and by continuous gene expression profiling of leaves at weekly intervals from transplanting until harvesting. Our gene expression profiling provides baseline information for functional characterization of genes revealed by the complete sequencing of the rice genome and for more exhaustive annotation of the elucidated genome. More importantly, we uncovered two drastic changes of leaf transcriptional programs reflecting growth stage-specific gene expression signatures that not only confirmed previously known physiological processes but also established new insights into developmental plant physiology that were never before demonstrated by studies involving non-global or semi-global approaches.

## Results and Discussion

### Generation of gene expression profiles covering various tissues and organs

**Figure 1:**
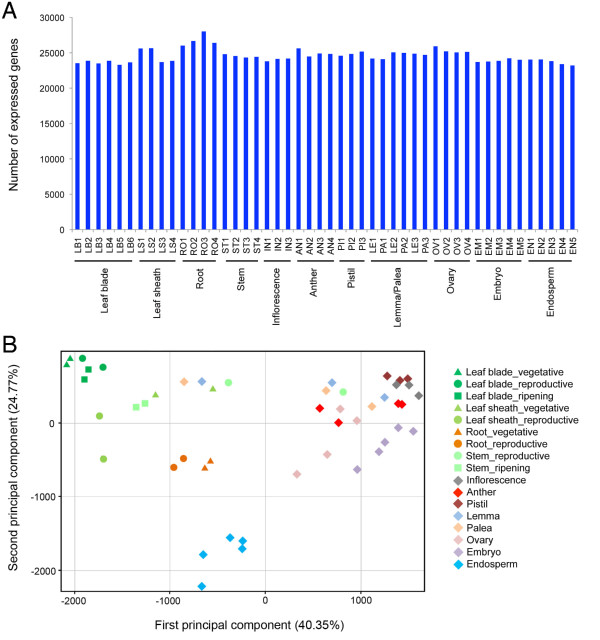
**Overview of gene expression profile of organs and tissues at various stages of growth **. (A) Number of expressed genes in each organ and tissue across the entire spatiotemporal developmental cycle. The genes with normalized signal intensities above -5 were extracted as 'expressed' genes. (B) PCA applied to the expression profiles of 48 samples identified organ/tissue-specific gene expression signatures. The average normalized signal intensities for each sample were used in this analysis.

We performed spatiotemporal gene expression profiling using 48 different tissue and organ types representing the entire growth and developmental cycle from transplanting to harvesting (Table 1; See Additional file 1). Samples for vegetative organs, such as leaf blade, leaf sheath, root, and stem, were obtained at midday (12:00) and midnight (24:00) at the vegetative, reproductive, and seed ripening stages with reference to the number of days after transplanting (DAT). The entire inflorescence and specific floral organs, such as anther, pistil, lemma, and palea, were collected at various developmental stages. After the onset of pollination, the ovary, embryo, and endosperm were sampled at 10:00 AM based on the number of days after flowering (DAF). Transcriptome analysis was performed with the Agilent 44K rice microarray, which contains 35,760 independent probes corresponding to 27,201 annotated loci published in RAP-DB [4]. We obtained a total of 143 microarray data representing triplicate expression profiles for each organ/tissue sample except for one sample of anther (Table 1). Correlation coefficients calculated for each of the replicates indicates that all but two were above 0.9, testifying to the quality of the expression data (See Additional file 2). The number of expressed genes across organs/tissues did not vary significantly and ranged from 63-76% (Figure 1A) and about 43% (15,224) of the transcripts were expressed in all organs/tissues. Principal component analysis (PCA) revealed three distinct transcriptome clusters corresponding to the profiles of vegetative organs such as leaf, stem, and root; reproductive organs such as anther, pistil, and entire inflorescence; and the endosperm (Figure 1B). The profiles of lemma and palea clustered together with the reproductive organs in earlier stages of development and with the vegetative organs at the later stages. Relative expression levels of Gene Ontology (GO) categories using samples from various organs/tissues at various developmental stages revealed that photosynthesis-related genes had high expression values in leaf blade, leaf sheath, stem, and lemma/palea at the later developmental stage, while cell proliferation-related genes had high scores in inflorescence, anther, pistil, and lemma/palea in the early developmental stage (See Additional file 3). The transcriptome profiles of the endosperm were quite different from the others (Figure 1B) and the GO categories related to glycogen biosynthesis showed high relative expression values, consistent with it being a specialized tissue for nutrition and storage.

**Table 1:** Samples used in spatiotemporal gene expression profiling

**No**.	Sample ID	Organ/Tissue	Sampling details	Replicate
1	LB1	Leaf blade	27 days after transplanting_12:00	3
2	LB2	Leaf blade	27 days after transplanting_24:00	3
3	LB3	Leaf blade	76 days after transplanting_12:00	3
4	LB4	Leaf blade	76 days after transplanting_24:00	3
5	LB5	Leaf blade	125 days after transplanting_12:00	3
6	LB6	Leaf blade	125 days after transplanting_24:00	3
7	LS1	Leaf sheath	27 days after transplanting_12:00	3
8	LS2	Leaf sheath	27 days after transplanting_24:00	3
9	LS3	Leaf sheath	76 days after transplanting_12:00	3
10	LS4	Leaf sheath	76 days after transplanting_24:00	3
11	RO1	Root	27 days after transplanting_12:00	3
12	RO2	Root	27 days after transplanting_24:00	3
13	RO3	Root	76 days after transplanting_12:00	3
14	RO4	Root	76 days after transplanting_24:00	3
15	ST1	Stem	83 days after transplanting_12:00	3
16	ST2	Stem	83 days after transplanting_24:00	3
17	ST3	Stem	90 days after transplanting_12:00	3
18	ST4	Stem	90 days after transplanting_24:00	3
19	IN1	Inflorescence	Inflorescence length 0.6-1.0 mm	3
20	IN2	Inflorescence	Inflorescence length 3.0-4.0 mm	3
21	IN3	Inflorescence	Inflorescence length 5.0-10.0 mm	3
22	AN1	Anther	Anther length 0.3-0.6 mm	2
23	AN2	Anther	Anther length 0.7-1.0 mm	3
24	AN3	Anther	Anther length 1.2-1.5 mm	3
25	AN4	Anther	Anther length 1.6-2.0 mm	3
26	PI1	Pistil	Pistil from 05-10 cm inflorescence	3
27	PI2	Pistil	Pistil from 10-14 cm inflorescence	3
28	PI3	Pistil	Pistil from 14-18 cm inflorescence	3
29	LE1	Lemma	Lemma from 1.5-2.0 mm floret	3
38	PA1	Palea	Palea from 1.5-2.0 mm floret	3
31	LE2	Lemma	Lemma from 4.0-5.0 mm floret	3
32	PA2	Palea	Palea from 4.0-5.0 mm floret	3
33	LE3	Lemma	Lemma from >7.0 mm floret	3
34	PA3	Palea	Palea from >7.0 mm floret	3
35	OV1	Ovary	Ovary at 1 day after flowering_10:00	3
36	OV2	Ovary	Ovary at 3 days after flowering_10:00	3
37	OV3	Ovary	Ovary at 5 days after flowering_10:00	3
38	OV4	Ovary	Ovary at 7 days after flowering_10:00	3
39	EM1	Embryo	Embryo at 07 days after flowering_10:00	3
40	EM2	Embryo	Embryo at 10 days after flowering_10:00	3
41	EM3	Embryo	Embryo at 14 days after flowering_10:00	3
42	EM4	Embryo	Embryo at 28 days after flowering_10:00	3
43	EM5	Embryo	Embryo at 42 days after flowering_10:00	3
44	EN1	Endosperm	Endosperm at 07 days after flowering_10:00	3
45	EN2	Endosperm	Endosperm at 10 days after flowering_10:00	3
46	EN3	Endosperm	Endosperm at 14 days after flowering_10:00	3
47	EN4	Endosperm	Endosperm at 28 days after flowering_10:00	3
48	EN5	Endosperm	Endosperm at 42 days after flowering_10:00	3

### Organ/tissue-specific gene expression

**Figure 2:**
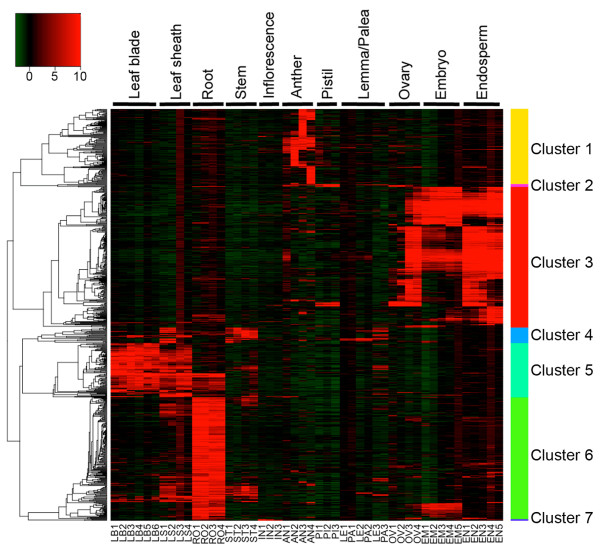
**Heat map of organ and tissue-specific expressed genes **. A total of 731 organ/tissue-specific genes identified by the Shannon entropy based method were analyzed by hierarchical clustering. A heat map was constructed using the relative expression values of the genes based on correlation distance and average linkage method. As a result, the 731 genes were grouped into 7 clusters based on organ/tissue-specificity of gene expression. High expression values are shown in red. Details of the samples used for each organ and tissue are described in Table 1.

The degree of a gene's specificity for a particular organ or tissue was estimated by the Shannon entropy scores [16], leading to the identification of 731 organ/tissue-specific genes corresponding to 660 loci (Figure 2; See Additional file 4). Nineteen percent of these genes were categorized as conserved hypothetical protein and hypothetical protein in the RAP-DB. We divided the organ/tissue-specific genes into 7 clusters based on the organ/tissue specificity of expression. The majority of the genes identified belonged to leaf- (Cluster 5), root- (Cluster 6), and seed- (Cluster 3) specific classes. Most of the genes specifically expressed in floral organs were found in anther (Cluster 1). Pistil- (Cluster 2), leaf sheath/stem- (Cluster 4), and inflorescence- (Cluster 7) specific genes belong to minor clusters, respectively.

Many seed-specific genes (Cluster 3) were expressed in both the embryo and endosperm or in the endosperm alone, while only a small number of genes showed embryo-specific expression (Figure 2). In addition, most of the seed-specific genes were induced from 5 days after flowering, when the embryo sac cavities are fully filled with endosperm cells and starch accumulation has been initiated, suggesting that most seed-specific genes are involved in grain filling and seed maturation. Among the 41 transcription factors showing organ or tissue-specific expression, 29 genes were seed-specific. These genes include *OsVP1*, which is an ortholog of the Arabidopsis *ABA insensitive 3 *(*ABI3*), and a homologue of *Leafy cotyledon 1 *(*LEC1*) (See Additional file 4), transcription factors that function in seed maturation [17]. The 29 seed-specific transcription factors contain 3 MADS-, 4 NAC-, 5 AP2-EREBP-, and 7 CCAAT-family genes. MADS-, NAC-, and CCAAT- family genes tend to express mainly in endosperm, and the expression of MADS genes were induced at early stages of seed development (from 1 to 14 DAF) in contrast with NAC and CCAAT genes which were expressed at the later stages (from 5 to 42 DAF) (See Additional file 5). On the other hand, AP2-EREBP genes were expressed mainly in embryo throughout seed development. These results suggested that each family of transcription factor might have a distinct function in embryo/endosperm development and grain filling. The seed-specific genes expressed at the early developmental stage include *SLRL2 *[18], a repressor of gibberellin (GA) signaling, and *OsETR2;2 *[19], a putative ethylene receptor that negatively regulates ethylene signaling, suggesting that repression of GA and ethylene signaling might also play a role in seed development.

The inflorescence specific genes (cluster 7) include *LAX PANICE *(*LAX*) and *FRIZZY PANICLE *(*FZP*). *LAX *gene encodes a putative transcriptional regulator containing a helix-loop-helix (bHLH) domain, which plays a role in axillary meristem formation [20,21] whereas FZP, an ERF transcription factor, represses the formation of the axillary meristem and establishes the spikelet meristem identity [22]. Differentiation of floral organs is more complex than other parts of the plant. Among them, the anther showed a unique feature in which most of the anther-specific genes (Cluster 1) were expressed only in a particular developmental stage (Figure 2). These results indicate the complex regulation of gene expression in both the gametophytic and sporophytic tissues during anther development [9]. Pollen-specific genes contained 4 transcription factors, one of which encodes Tapetum Degeneration Retardation (TDR), a basic helix-loop-helix (bHLH) transcription factor [23]. TDR is a putative ortholog in rice of ABORTED MICROSPORES (AMS) in Arabidopsis, which play a role in tapetal cell development and postmeiotic microspore formation [24], and has recently been reported to interact with two bHLH proteins, AtbHLH089 and AtbHLH091 [25]. Os04g0599300 encodes a close homolog of *AtbHLH089 *and *AtbHLH091*, and is also involved in pollen-specific expressed genes, implying that in rice TDR would interact with the bHLH protein encoded by Os04g0599300 as in Arabidopsis. These results suggest that a wide range of expression profiling can be very useful as well in elucidating the interactome in cereal crops. In comparison with the anther, only a few specific genes were identified in the pistil (Cluster 2), where megasporogenesis and megagametogenesis occur. This is probably because rice has a monocarpellary ovary with a single ovule and transcripts associated with such events may be masked.

To characterize the expression profile of lemma and palea, we performed a two-way analysis of variance (ANOVA; FDR < 0.05) using tissues (lemma/palea) and the sizes corresponding to the developmental stages as factors. The two-way ANOVA identified 23 genes that were differentially expressed between lemma and palea, irrespective of the developmental stages, while 20,007 genes showed differential expression among the stages, irrespective of the tissues. None of genes showed interaction between the two factors. The results implied that lemma and palea have similar transcriptome profile as predicted from the similar morphology and function. However, among the 23 genes, 13 genes encode transcription factors including *DROOPING LEAF *[26] and *MOSAIC FLORAL ORGANS1/OsMADS6 *[27] which were expressed specifically in the lemma and palea, respectively (See Additional file 6), suggesting that these transcription factors maybe key regulators in the differentiation of lemma and palea.

### Diurnal and growth stage-specific gene expression

**Figure 3:**
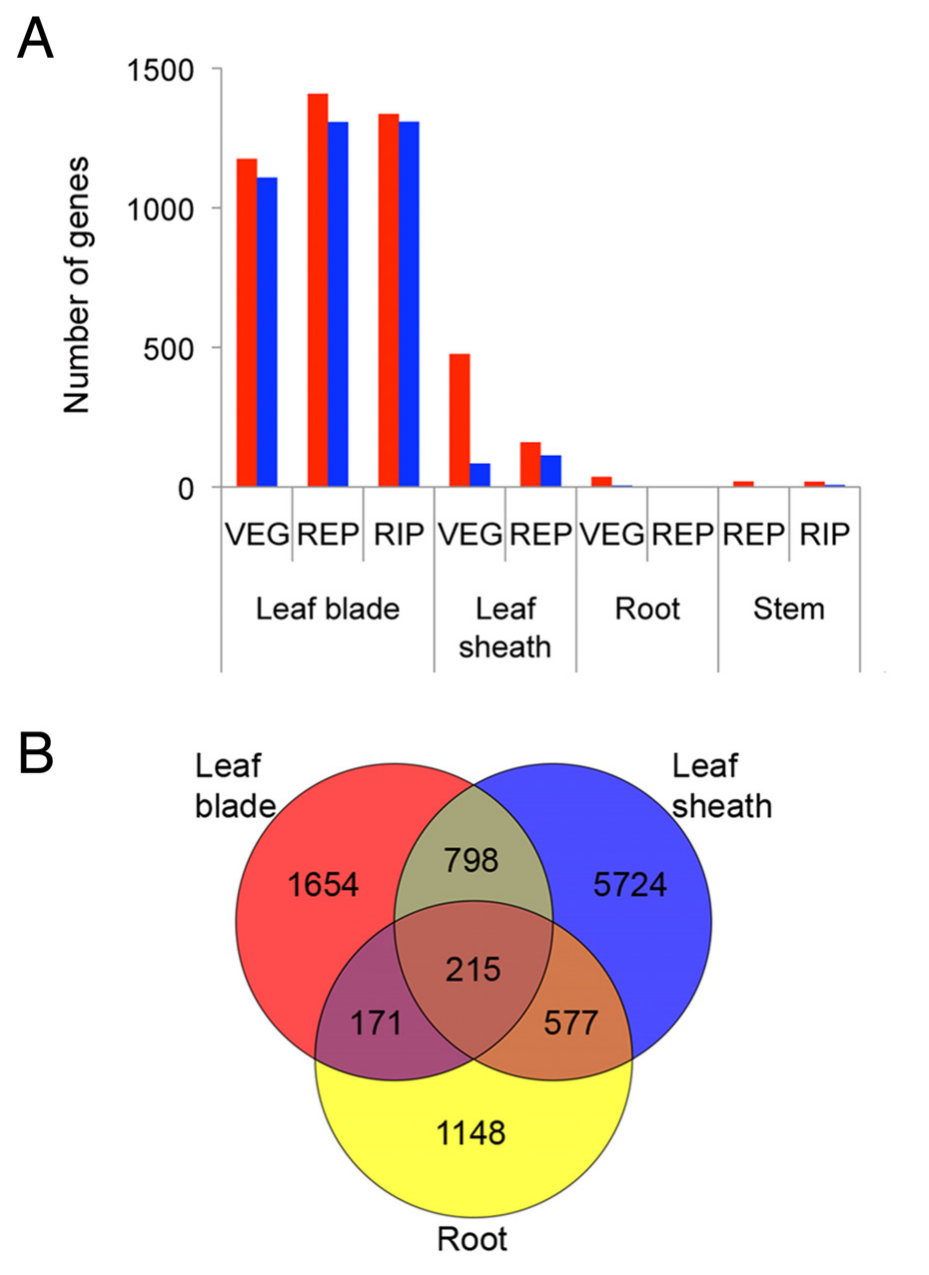
**Diurnal and growth stage-specificity of gene expression **. (A) Frequency of diurnally expressed genes in the vegetative organs. Genes differentially expressed between daytime (12:00) and nighttime (24:00) were extracted based on the t-test and fold change criteria (FDR < 0.05 and fold change, FC > 3) in each organ/tissue. Red and blue bars represent highly expressed genes at daytime and nighttime, respectively. (B) Venn diagram of differentially expressed genes from the vegetative to reproductive phases in leaf blade, leaf sheath, and root during daytime. The differentially expressed genes were statistically extracted based on the t-test and fold change criteria (FDR < 0.05 and FC > 3).

The transcriptomes of vegetative organs at daytime and nighttime showed diurnal patterns for about 7% of transcripts particularly in mature leaf blade (Figure 3A). The number of genes with diurnal expression pattern was much less in leaf sheath, and rare in root and stem, reflecting the importance of diurnal regulation of gene expression in the leaf blade for its biological functions such as photosynthesis. At the vegetative stage, a total of 20 genes were universally expressed with a diurnal pattern in leaf blade, leaf sheath, and root, including circadian-associated genes [28], *OsPRR95 *and *OsPCL1*, which showed high expression values at daytime and nighttime, respectively (See Additional file 7). Although diurnal expression depends on the daily rhythm induced by the light/dark cycle, several genes including the circadian-associated genes are also diurnally regulated in the root, which is not exposed to light under field conditions. It was recently reported that in Arabidopsis the circadian clock of the root is different from that of the shoot and is synchronized by a photosynthesis-related signal from the shoot [29].

In the leaf blade, leaf sheath, and root, the expression of many genes also showed growth-stage specific signatures. We extracted 215 genes that universally showed changes in expression in all 3 of these tissues from vegetative to reproductive stages (Figure 3B; See Additional file 8). These genes included four MADS box transcription factors, *OsMADS1*, *OsMADS14*, *OsMADS15*, and *OsMADS18*, which were highly expressed in the reproductive phase. *OsMADS14 *and *OsMADS15 *are homologs to an Arabidopsis floral identity gene *APETALA1*, and were reported to be induced by *Hd3a *and *RFT1*, rice orthologs of Arabidopsis florigen gene *FLOWERING LOCUS T *(*FT*) [30,31]. Hd3a and RFT1 are synthesized in leaf blade and transported to the shoot apical meristem (SAM) through phloem as a florigen [31,32]. Although the expression of *OsMADS14 *and *OsMADS15 *may not be directly affected by Hd3a and RFT1 particularly in roots, the transition from vegetative to reproductive phase may have induced the changes in the transcriptome of vegetative organs resulting in the expression of such reproductive organ identity genes. Among the universally downregulated genes going from the vegetative to reproductive phase, we also found a number of phosphate (Pi)-starvation induced genes which may be related to the physiological state transition associated with the reproductive phase change as discussed below.

### Continuous gene expression profiling throughout the entire growth cycle in the field

**Figure 4:**
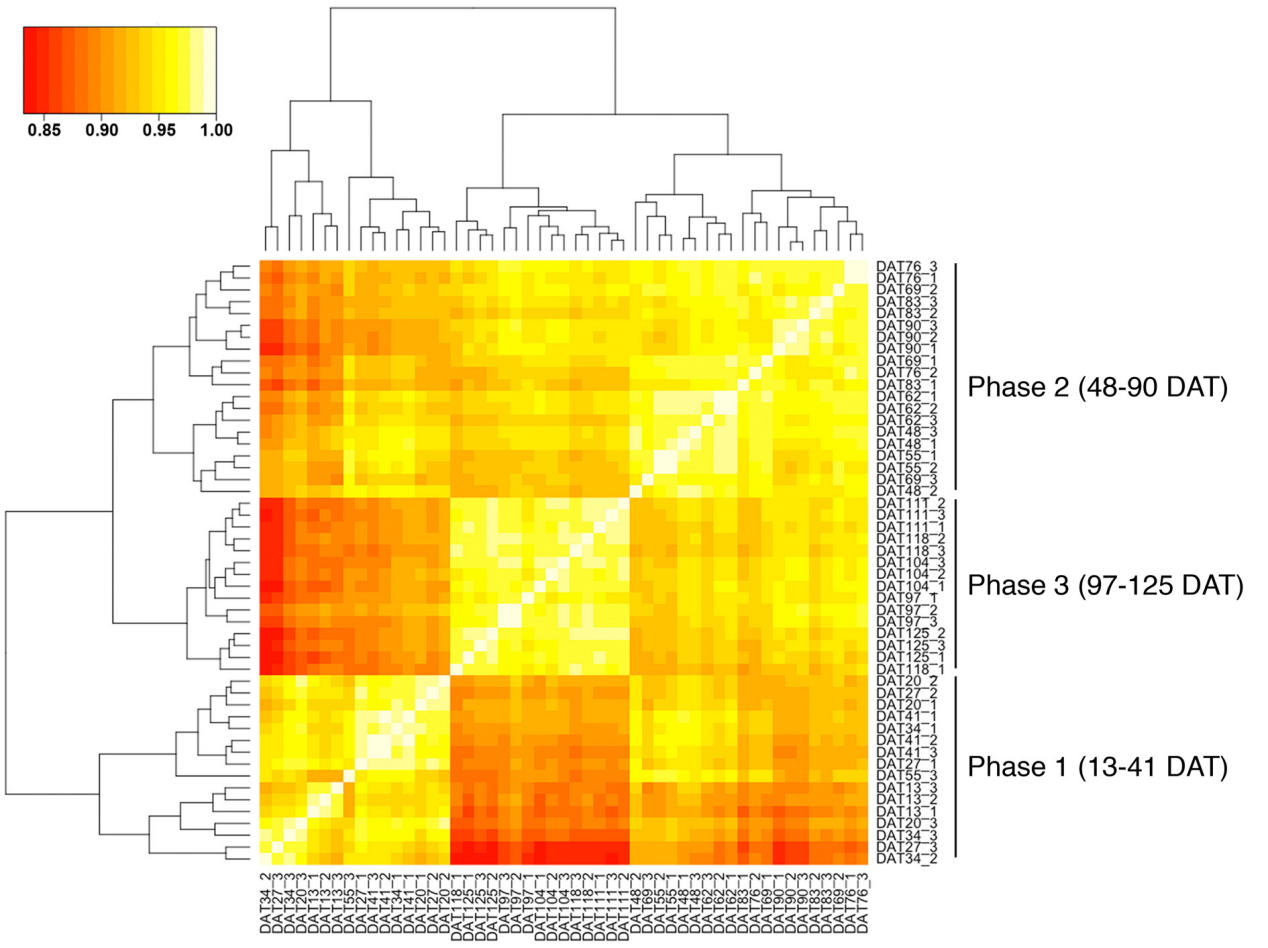
**Correlation of expression profiles of the leaf from 13 to 125 DAT **. Pearson's correlation coefficients (PCCs) were calculated using the normalized signal intensities of the 29,119 genes. Samples were clustered based on Euclidian distance and complete linkage. Transcriptome profiles were apparently grouped into phase 1 (13-41 DAT), phase 2 (48-90 DAT), and phase 3 (97-125 DAT) corresponding approximately to vegetative, reproductive, and ripening stages of growth, respectively. The color scale represents the PCC scores. DAT: days after transplanting.

In order to further understand the transcriptional programs associated with growth stage of rice grown under the natural field conditions, we performed continuous gene expression profiling of the leaves from 13 until 125 DAT in 2008 to establish a transcriptome profile encompassing the entire growth phase in the field. The uppermost fully-expanded leaf in the main stem, representing the 1st leaf up to 76 days after transplanting (DAT) and the flag leaf from 83 DAT until harvesting, were sampled at 12:00 PM every 7 days, covering 17 different growth stages with three replicates (See Additional file 1). For analyses, we used 29,119 probes with raw signal intensities above 100 in at least one sample of all 51 expression profiles. Interestingly, Pearson's correlation coefficients (PCCs) calculated across the 51 expression profiles identified three phases with high PCC scores, namely, 13-41 DAT (phase 1), 48-90 DAT (phase 2), and 97-125 DAT (phase 3) which approximately correspond to the vegetative, reproductive, and ripening stages, respectively (Figure 4). These results suggested that two major transcriptome changes occurred in the leaves from transplanting until harvesting.

### First major transcriptome change associated with reproductive transition

**Figure 5:**
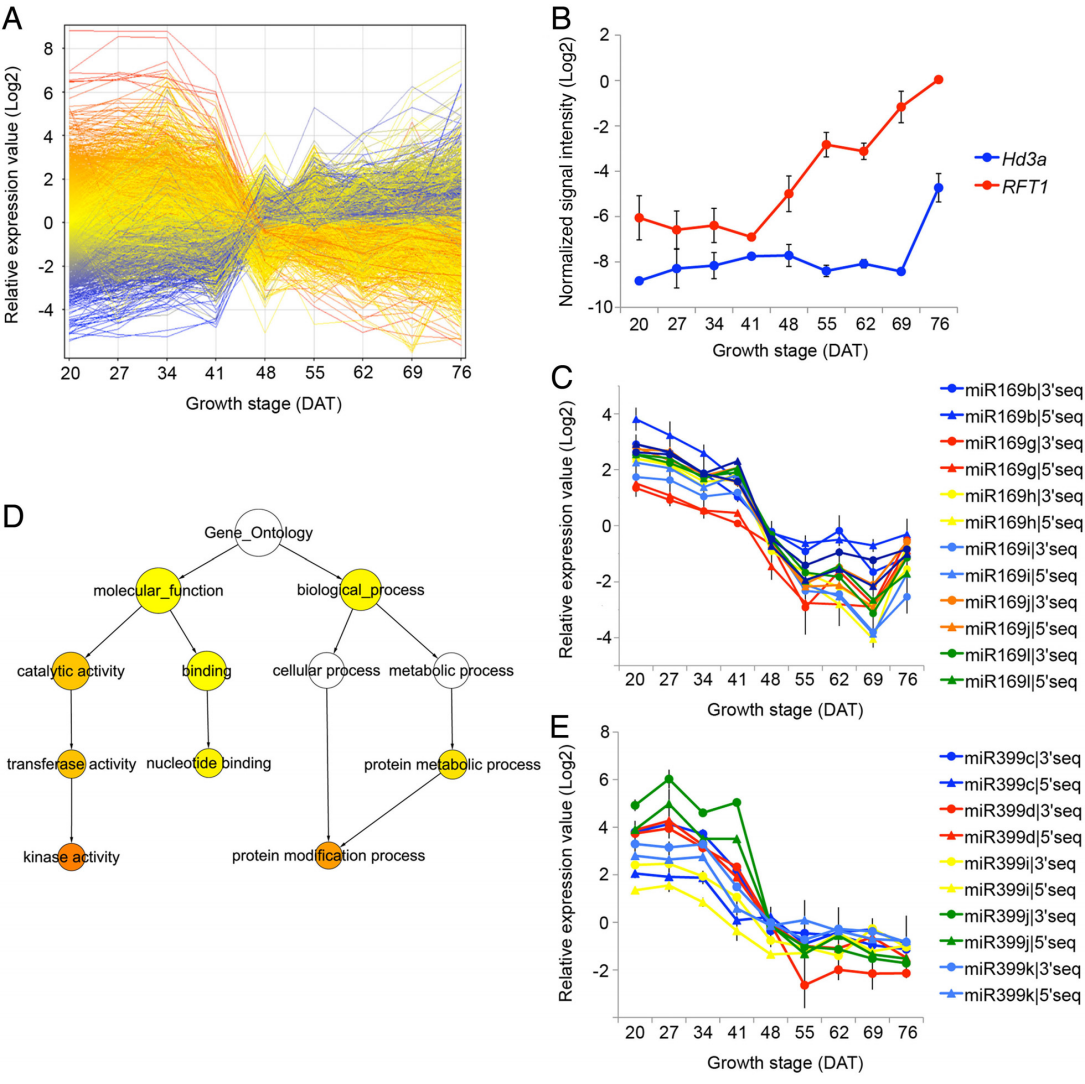
**Change in transcriptome associated with the transition to reproductive stage **. (A) Expression pattern of 29,119 genes from 20 to 76 DAT based on relative expression values indicate drastic change between 41-48 DAT. Blue, yellow, and red lines indicate high, middle, and low expression values, respectively, at 20 DAT. (B) Expression pattern of rice florigen genes, *Hd3a *(blue) and *RFT1 *(red), from 20 to 76 DAT. Error bars indicate s.e.m. (n = 3). (C) Expression pattern of seven miR169 precursors from 20 to 76 DAT. Each miRNA precursor was represented in the microarray as two probes corresponding to the 3' and 5' sequence, respectively. Error bars indicate s.e.m. (n = 3). (D) GO analysis of the 357 genes upregulated from 41 to 48 DAT. The colored circles represent enriched categories based on the p-values corrected for multiple testing (FDR) ranging from 0.05 (yellow) or below (orange). The size of the circle is proportional to the number of genes annotated to that node. (E) Expression pattern of five miR399 precursors from 20 to 76 DAT. Error bars indicate s.e.m. (n = 3).

The first major change observed between phase 1 and phase 2 was assumed to be associated with the transition from vegetative to reproductive stage. The expression profile based on the relative expression values of 29,119 genes showed that a drastic change in leaf transcriptome occurred between 41 DAT and 48 DAT (Figure 5A). A similar change was confirmed in 2009 and in the leaves below including the 2nd, 3rd, 4th and 5th leaves on the basis of PCA (See Additional file 9). At 56 DAT, approximately 50% of rice plants in the field were in initiation of panicle development, and at 58 DAT most plants examined were already in the early stage of panicle development, indicating that the drastic change in the leaf transcriptome occurred before the initiation of panicle development. While *Hd3a *was not induced until 69 DAT when the young panicle was completely differentiated, *RFT1 *was induced as early as 48 DAT (Figure 5B). This suggests that induction of flowering might be controlled by RFT1 in the natural conditions in Tsukuba, Japan (~36°N), where natural day length at the time of reproductive transition is under long-day (LD) conditions. Consistent with this observation, Hd3a reportedly functions as a mobile flowering signal in short-day (SD) conditions while RFT1 functions in LD [31]. In addition, Itoh et al. [33] reported that the critical day length for *Hd3a *expression was around 13.5 h further supporting the fact that *Hd3a *was not induced before the reproductive transition in our field conditions.

We observed the reduction of miR169 precursors at the first transcriptome change (Figure 5C; See Additional file 10). The target of miR169 is the HAP2 type transcription factor (also as known as NF-YA), which is thought to be involved in various traits, e.g., flowering and drought tolerance in Arabidopsis [34,35], and nodule development in *Medicago truncatula *[36]. Ten *HAP2 *genes have been identified in rice [37]. The expression of six *HAP2 *genes with the predicted miR169 target sites in their 3' UTRs (*OsHAP2C*, *D*, *E*, *F*, *G*, and *H*) increased in the first transcriptome change, but those of two *HAP2 *genes without a target site (*OsHAP2A *and *OsHAP2B*) did not change (See Additional file 10), suggesting the function of miR169 in the regulation of *HAP2 *expression in the first major transcriptome change. In Arabidopsis, CONSTANS (CO), which contains a CCT domain, is the key regulator of flowering genes [38,39]. The CCT domain exhibits similarity to a domain of HAP2, which mediates the formation of the HAP trimeric complex, HAP2/HAP3/HAP5. It has been suggested that replacement of CO with AtHAP2 in the HAP trimeric complex by overexpression of *AtHAP2 *delays flowering via down-regulation of FT [34]. In SD-flowering rice plants, the CCT-domain containing proteins Hd1 and Ghd7 regulate flowering by repressing expression of the florigen genes in LD [40-42]. Wei et al. [43] has reported that *DTH8 *QTL for days-to-heading encodes a putative HAP3 subunit for the trimeric HAP2/HAP3/HAP5 complex and suppresses flowering in LD, and further speculated that the formation of the Hd1/DTH8/HAP5 and Ghd7/DTH8/HAP5 complex might be associated with the suppression of flowering by the downregulation of *Ehd1 *and *Hd3a *in LD. In this scenario, increased expression of *OsHAP2 *caused by miR169 reduction promotes reproductive transition in rice through functional inhibition of the CCT-domain containing proteins and the resultant induction of *RFT1 *expression, which was observed at the first major transcriptional change. Three of the six *HAP2 *genes were universally upregulated in root as well as leaf from vegetative to reproductive stages (See Additional file 8). In plant, HAP system has been thought to play diverged roles in gene transcription because each subunit in HAP complex, HAP2/HAP3/HAP5, represents a gene family [37,44]. For example, it has been reported that NFYA5, a HAP2 type transcriptional factor regulated by miR169, is important for drought resistance in Arabidopsis [35]. Therefore, miR169-mediated *HAP2 *genes expression might synchronously regulate not only flowering time but also other agronomically important traits such as resistance to biotic and abiotic stress.

We extracted 1,316 genes with different expression patterns at 41 DAT and 48 DAT. A total of 357 upregulated and 333 downregulated genes were then selected based on their similarity in expression patterns from the results of hierarchical cluster analysis (See Additional file 11 and 12). The upregulated genes comprised a large number of 'newly expressed' genes, which were hardly detectable at 34 and 41 DAT (See Additional file 11). Gene Ontology (GO) analysis showed that the genes encoding protein kinase were significantly enriched among the upregulated genes (Figure 5D). The results indicated that many signal transduction pathways accompanied by protein phosphorylation processes participate in the transition between phase 1 and phase 2. A number of genes that are induced under Pi-starvation conditions were downregulated from 41 to 48 DAT [45,46]. In Arabidopsis, regulation of miR399 and the ubiquitin-conjugating E2 enzyme gene *PHO2 *plays a central role in the maintenance of Pi homeostasis [47,48]. miR399 generated in shoots serves as a long-distance signal that represses *PHO2 *in roots under Pi-starvation conditions, resulting in activation of Pi uptake and translocation [49,50]. Five precursors of miR399 were downregulated in leaves before the initiation of panicle development and the potential rice ortholog, *OsPHO2 *[47], was upregulated in roots, suggesting an alteration of Pi homeostasis at this stage (Figure 5E; See Additional file 13). *MGD2 *and *MGD3 *encode type-B monogalactosyldiacylglycerol (MGDG) synthase and are involved in Pi-starvation induced lipid remodeling for Pi-recycling, a typical response of Pi starvation [51]. Os08g0299400, a close homolog of *MGD2 *and *MGD3 *of Arabidopsis, was 225.4-fold downregulated from 41 DAT to 48 DAT, consistent with the relaxation of Pi demand described above. PHR1 is a key transcriptional activator in controlling Pi uptake and allocation, and the PHR1 binding motif is often found in the upstream regions of Arabidopsis genes induced by Pi-starvation [52]. The PHR1 binding motif was enriched in the 1-kb upstream regions of the 333 downregulated genes, further supporting the alteration of Pi homeostasis (See Additional file 14). The expression of many Pi-response genes was changed in leaf sheath and root as well as leaf blade in the transition from the vegetative to reproductive phases (See Additional file 8). These observations strongly suggest that the rice plant undergoes a change in Pi homeostasis at the vegetative-reproductive phase transition. Pi is an important nutrient for increasing the number of tillers, one of the components of grain yield. The high demand for Pi during vegetative stage may be vital for proper development of tillers and rice plants may not need much Pi after the reproductive-phase transition, when few tillers are produced.

Taken together, the first transcriptome change involves not only the initiation of panicle development but also various aspects of the physiological state, which might be prerequisite for proper flowering and later developmental stages. The drastic phase change in shoot apical meristem is initiated by long-distance transport of FT family protein synthesized in leaves. Our results suggest that changes in physiological state also occurred in other tissues and organs, at least at the same time of induction of FT-like gene expression in leaves under the natural field conditions, and revealed interesting trends suggesting the potential role of similar or related signaling events in mediating the transcriptome change. One possibility is that floral transition and the shift in Pi homeostasis are parallel consequences of the same signaling event. Another is that the transcriptomes associated with Pi homeostasis and floral transition were consequences of independent signaling that happened to be developmentally coincident of each other. Although plant development is thought to be a continuous process, the phase transition maybe characterized by a transcriptome which is distinguishable from both the vegetative and reproductive phases. Further studies using various growth conditions as well as various cultivars and mutant lines maybe necessary to clarify the machinery of phase change.

### Second major transcriptome change associated with senescence

**Figure 6:**
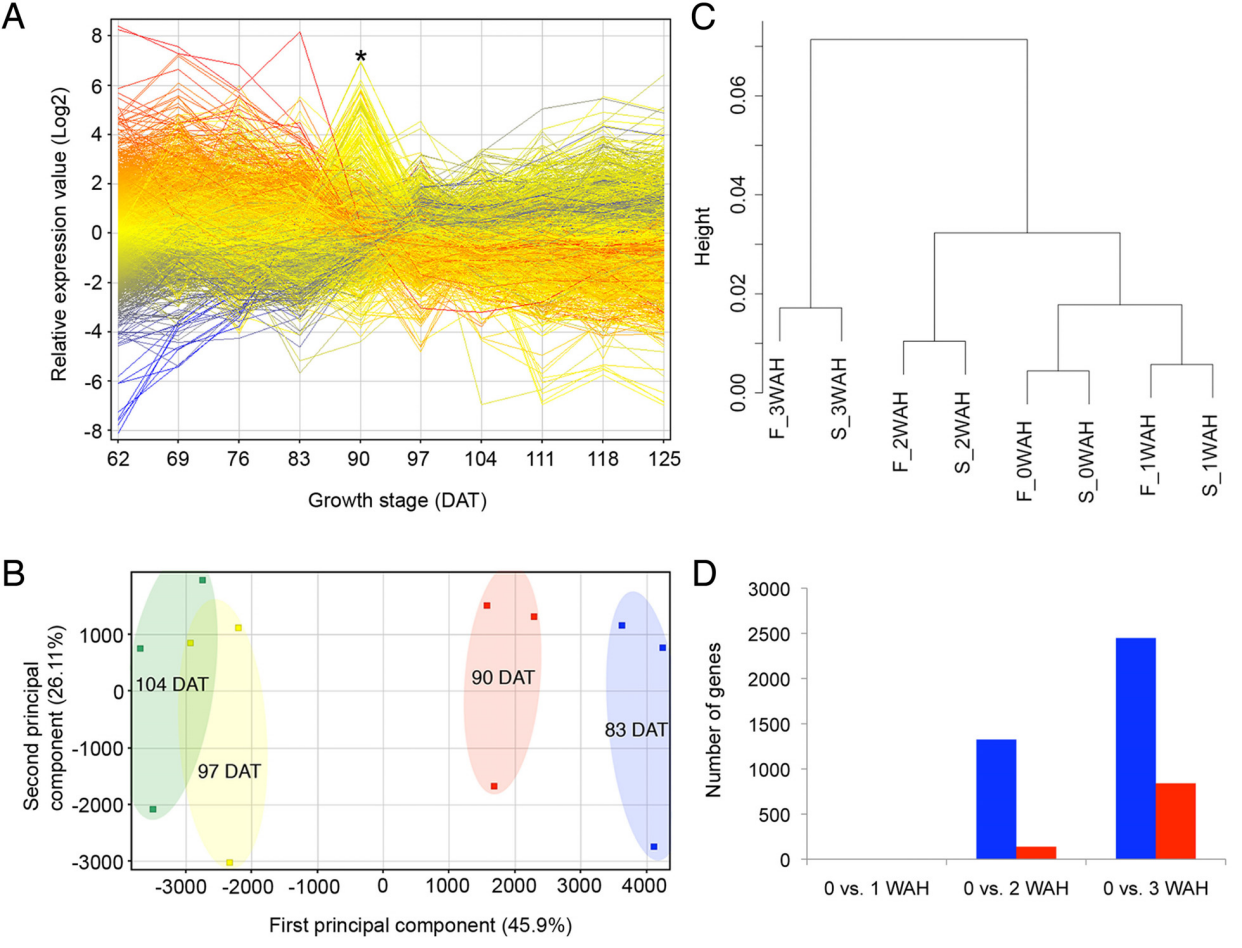
**Change in transcriptome associated with flowering and grain filling **. (A) Expression pattern of 29,119 genes from 62 to 125 DAT based on relative expression values. Transient expression of pollen specific genes at 90 DAT (indicated by asterisk), which also coincided with the peak of flowering, was due to pollen contamination of leaf samples (See Additional file 15). (B) PCA of the expression profile of flag leaf at 83, 90, 97 and 104 DAT. (C) Cluster analysis of fertile (F) and sterile (S) plants at 0, 1, 2 and 3 weeks after heading (WAH) based on correlation distance and complete linkage. (D) Differentially expressed genes in flag leaf of fertile (blue) and sterile (red) lines after heading were statistically extracted based on t-test and fold change criteria (FDR < 0.05 and FC > 2).

Next, we focused on the leaf expression profiles from 62 DAT to 125 DAT to examine the second major transcriptome change observed at the transition between phase 2 and phase 3. The expression profile of 29,119 genes revealed that the change in transcriptome occurred around 90 DAT (Figure 6A), when most of the rice plants in the field were at various stages of flowering. Eighty genes showing very high and transient expression at 90 DAT were pollen-specific genes, suggesting contamination of the leaf samples by pollen dispersed during anthesis (See Additional file 15). PCA excluding these genes revealed that the transcriptome change mainly occurred between 90 DAT and 97 DAT, the start of the post-flowering process, i.e., seed development (Figure 6B). We extracted differentially expressed genes including 423 upregulated and 573 downregulated genes between 83 DAT and 97 DAT (See Additional file 16). Among the 423 upregulated genes, six NAC transcription factors were identified (See Additional file 16), one of these (Os07g0566500) is a close homolog of wheat NAM-B1, which was isolated as a QTL gene accelerating senescence and increasing nutrient remobilization from leaves to developing grains [53]. *OsNAP *(Os03g0327800) is a close homolog of *AtNAP*, of which a loss-of function mutation is known to result in a delay of leaf senescence in Arabidopsis [54]. These results suggest that the second transcriptome change is associated with leaf senescence, an active process whereby nutrients are salvaged from senescent leaves for use by emerging leaves and reproductive organs. To examine the role of formation of a very strong sink, i.e., developing seeds, in the second transcriptome change, we performed expression profiles on three independent sterile-mutant lines, *pair1 *[55], *pair2 *[56,57], and *mel1-1 *[58], in 2009 (See Additional file 17). The fertile and sterile lines basically showed similar expression profiles at the same sampling time, but the transcriptome change in the fertile lines was more rapid and enhanced than that of the sterile lines (Figure 6C and 6D). This result indicates that the second major transcriptome change is associated with leaf senescence, which autonomously starts independent of the development of the sink, but is accelerated by the sink formation. Delaying leaf senescence in order to maintain the photosynthetic activity for as long as possible may improve source potential. However, we noted that the expression of photosynthesis related genes as described in KEGG database [59], namely, osa00196 (Photosynthesis-antenna proteins) and osa00195 (Photosynthesis), decreased more drastically in fertile plants than in sterile plants (See Additional file 18), suggesting that the process of nutrient translocation has a negative effect on photosynthetic potential in senescent leaves. It is therefore unlikely that delaying leaf senescence is a viable approach for improving source potential in rice. In contrast with the QTLs for sink size [60-62], the QTL gene associated with source potential has not yet been cloned, presumably due to the complexity of sink-source interaction which makes it difficult to monitor physiological traits associated with photosynthesis and nutrient translocation. We have shown here particularly in the analysis of the first transcriptome change that a wide range of transcriptome profile could provide new insights into many physiological processes that underlie phase transition. Therefore, further high-resolution transcriptome analysis and data mining for integrative physiology will be essential in elucidating the complex regulation of sink-source interaction.

## Conclusions

By spatiotemporal gene expression profiling, we were able to clarify organ/tissue-specific, diurnal, and growth stage-specific gene expression signatures throughout the entire growth cycle under natural field conditions. Our analysis also highlights the synchronized change of gene expression across separate organs and tissues suggesting the possible involvement of a long distance signaling mechanism in developmental and diurnal gene expression. Consistent with this, we found that rice florigen gene and miR399 expressed in leaves have direct effects on gene activity in other organs. More importantly, we have identified two major changes of leaf transcriptional programs reflecting growth stage-specific gene expression signatures. The drastic change in phosphate-homeostasis state before the panicle differentiation as evident from the behavior of several key regulators such as miR399 under the natural field conditions maybe associated with reproductive processes involved in expression of agronomic traits that determine crop yield. Taken together, a field transcriptome obtained by a wide range of gene expression profiling provided not only baseline information for functional characterization of genes but also revealed critical developmental and physiological transitions involved in the expression of growth potential under natural field conditions. With the accompanying gene expression profile database, RiceXPro [63]http://ricexpro.dna.affrc.go.jp/, our resources on gene expression profiling may contribute to innovative crop improvements that have not yet been tried in classical or molecular breeding.

## Methods

### Plant materials and sampling

Rice (*Oryza sativa *L. ssp. *japonica *cv. Nipponbare) seeds were sown in germinating boxes. At 30 days after germination, the seedlings were transplanted in a paddy field in Tsukuba, Japan (~36°N) and grown under normal conditions during the 2008 cultivation season. To clarify the effect of sink formation during leaf senescence, three sterile *Tos17 *insertion mutant lines http://tos.nias.affrc.go.jp/~miyao/pub/tos17/, namely, *pair1 *(ND0016) [55], *pair2 *(NC0122) [56,57], and *mel1-1 *(NC0005) [58], were also grown in the field in 2009 for leaf sampling. Fertile plants (homozygous or heterozygous) derived from a segregating population were used for comparison. All samples were immediately frozen in liquid nitrogen and stored at -80°C until RNA isolation.

### Microarray analysis

Total RNA was isolated from each plant sample using RNeasy plant mini kit (QIAGEN). For endosperm samples, total RNA was isolated by phenol extraction [64], and further purified using the mini spin column from the RNeasy plant mini kit. The extracted RNA was quantified using NanoDrop ND-1000 UV-VIS spectrophotometer (NanoDrop) and checked for quality using an Agilent 2100 Bioanalyzer (Agilent Technologies, Palo Alto, CA, USA). One-color spike-mix was added to the total RNA prior to the labeling reaction. Labeling was performed using a Quick Amp Labeling Kit, One-Color (Agilent Technologies) in the presence of cyanine-3 (Cy3)-CTP according to the manufacturer's protocol. For microarray hybridization, 1650 ng of Cy3-labeled cRNA (except for samples with low cRNA yield) was fragmented and hybridized on a slide of rice 4 × 44K microarray RAP-DB (Agilent; G2519F#15241) at 65°C for 17 hours. Hybridization and washing of the hybridized slides were performed according to the manufacturer's instruction. Slides were scanned on the Agilent G2505B DNA microarray scanner, and background correction of the Cy3 raw signals was performed using the Agilent Feature Extraction software (version 9.5.3.1).

### Statistical analysis

The processed raw signal intensity of all probes (45,151) were subjected to 75 percentile normalization with GeneSpringGX11 for inter-array comparison (Agilent Technologies) and transformed to log2 scale. A total of 35,760 probes were extracted after the normalization and used for analysis of the gene expression profile of various organs/tissues from 143 microarray data. For comparison of expression patterns of each gene, we performed an additional normalization procedure. The median expression values across the data used for each analysis were subtracted for each gene. The gene-normalized values were assigned as relative expression values. Analysis of the continuous expression profiles comprising of 51 microarray data, was based on 29,119 probes with raw signal intensities above 100 in at least one sample. Unpaired t-test and PCA were performed with the GeneSpringGX11. In the t-test, the p values were adjusted for multiple testing by the Benjamine and Hochberg's method to correct the false discovery rate (FDR). For hierarchical cluster analyses in Additional file 11 and 16, we used the uncentered Pearson correlation and centroid linkage algorithm with relative expression values with the GeneSpringGX11. We performed statistical analysis of sterile and fertile lines using expression profiles of ND0016, NC0122, and NC0005 as three replicates. Analysis was based on 24,577 probes with raw signal intensities above 100 in at least one sample. The heat maps in Figure 2, 4, Additional file 3, and 5 were constructed using heatmap.2 in the "gplots" package of R program http://www.R-project.org. Clustering of fertile and sterile lines in Figure 6C were performed based on correlation distance and complete linkage with normalized signal intensities of 24,577 probes using the R program.

### Extraction of organ/tissue-specific expressed genes

We used Shannon entropy to evaluate the organ and tissue specificity of gene expression [16]. The raw signal intensity values were applied to quantile normalization and transformed to log2 scale with GenespringGX11 prior to calculation of Shannon entropy. We selected a total of 731 organ/tissue-specific genes with entropy values below 4.5 and relative expression values above 8 in at least one sample. Transcription factors contained in the organ/tissue-specific genes were extracted based on the information in PlnTFDB [65]

### Analysis of miRNA expression

In addition to the 35,760 probes, the rice 4x44K microarray RAP-DB contains 340 independent probes for the precursors of microRNAs with two different probes designed for each precursor. We used these probes to examine the expression of miR169 and miR399.

### GO analysis

For the GO analysis, generic GO annotated in RAP-DB was converted to GO slim using map2slim.pl script available from the go-perl page at CPAN http://search.cpan.org/~cmungall/go-perl/. For analysis of significantly enriched GO categories, we used the BiNGO plugin http://www.psb.ugent.be/cbd/papers/BiNGO/ for Cytoscape http://www.cytoscape.org/ with the default setting [66].

### Accession number

The data discussed in this study have been deposited in NCBI's Gene Expression Omnibus (GEO) [67], and are accessible through GEO Series accession number GSE21494.

## Authors' contributions

YS participated in the design of the research, and carried out the microarray analysis and the statistical analysis and wrote the manuscript. BA performed the microarray analysis and analysis the data and wrote the manuscript. NN performed statistical analysis and constructed the database. RM carried out RNA isolation and microarray analysis. KS performed sampling of endosperm and embryo and extracted the total RNA. HT carried out sampling of leaves in 2009 and the microarray analysis. HM and KK performed data analysis and constructed the database. MK performed the data analysis of sterile lines and helped edit the manuscript. HH participated in the design of the study and helped to draft the manuscript. YN conceived the project and designed the research. All authors read and approved the final manuscript.

## Supplementary Material

Additional file 1 **Overview of organ/tissue sampling performed to establish a field transcriptome of rice**. Plant organs/tissues were sampled at various stages of the developmental cycle. Vegetative organs such as leaf blade, leaf sheath, root and stem were sampled at three specific points corresponding to the vegetative, reproductive and ripening stages recorded as number of days after transplanting (DAT) at both daytime (12:00) and nighttime (24:00). Sampling for different stages of development of inflorescence and anther was based on the length of the inflorescence and the anther itself, respectively, whereas sampling for pistil and lemma/palea was based on the length of the inflorescence and the floret, respectively. Sampling for ovary, embryo and endosperm was based on the number of days after flowering (DAF). For continuous gene expression profiling, the uppermost leaf in the main stem was sampled at weekly intervals from 13 to 125 DAT. Approximately 50% of the rice plants observed in the field at 56 DAT were at panicle initiation stage. At 58 DAT, almost 90% of the plants were at the early stage of panicle development indicating a complete reproductive transition. Then by 90 DAT, all plants were either in the flowering stage or early stages of seed development corresponding to the ripening stage transition. Click here for file

Additional file 2 **Distribution of correlation coefficients calculated between biological replicates**. The correlation coefficients obtained for three replicates in most samples were above 0.9, except for two stem samples suggesting high quality of gene expression data. Click here for file

Additional file 3 **Hierarchical clustering of gene expression based on Gene Ontology categories**. The average expression value for all genes under the same GO category was used to analyze relative gene expression level. All GO terms with relative expression level above 2.5 in at least one sample were extracted and hierarchical cluster analysis was performed using the R program. Click here for file

Additional file 4 **Organ/tissue-specific genes**. Click here for file

Additional file 5 **Expression profile of seed-specific transcription factors**. Heat map of transcription factors highly expressed in developing seed, embryo and endosperm was constructed using the normalized signal intensity for each gene. High expression value is shown in red. Click here for file

Additional file 6 **Differentially expressed genes between lemma and palea**. Click here for file

Additional file 7 **Genes with common diurnal expression in vegetative organs**. Click here for file

Additional file 8 **Genes commonly regulated in vegetative organs from vegetative to reproductive phase**. Click here for file

Additional file 9 **Confirmation of the first major transcriptome change **(a) Expression profile of leaf at weekly from 20 DAT to 62 DAT during the 2009 cultivation season. Microarray analysis was performed with two replicates in each point. The first transcriptome change and panicle differentiation was observed earlier as compared to the 2008 cultivation season. (b) Changes in expression level of *Hd3a *and *RFT1*. The red line represents *RFT1 *and the two blue lines represent the two probes for *Hd3a*. (c) Changes in expression level of five miR399 precursors. (d) PCA of the gene expression profile at 41, 48, and 62 DAT during 2008 cultivation season based on the uppermost leaf (1^st ^leaf) in the main stem and the leaves below designed as the 2^nd^, 3^rd^, 4^th^, and 5^th ^leaf from the uppermost leaf. Distinct clustering of the gene expression profiles at various positions supported the transcriptome change observed from 41 to 48 DAT. The number in each cluster represent the leaf position in the main stem. Click here for file

Additional file 10 **Expression profile of miR169 and its target gene, *OsHAP2***. (a) Changes in expression of 16 miR169 precursors from 20 to 76 DAT. Error bars show s.e.m. (n = 3). (b) Changes in expression of 8 *OsHAP2 *genes from 20 to 76 DAT. HAP genes without the miR169 target sites (*OsHAP2A *and *OsHAP2B*) did not show change in expression. Error bars represent s.e.m. (n = 3). The corresponding RAP-DB loci are as follows: *OsHAP2A*, Os08g0196700; *OsHAP2B*, Os12g0613000; *OsHAP2C*, Os03g0174900; *OsHAP2D*, Os03g0696300; *OsHAP2E*, Os03g0411100; *OsHAP2F*, Os12g0618600; *OsHAP2G*, Os07g0608200; *OsHAP2H*, Os03g0647600. Click here for file

Additional file 11 **Differentially expressed genes between 41 and 48 DAT**. (a) Hierarchical cluster analysis was performed on relative expression values of all samples from 20 to 76 DAT. A total of 1,316 differentially expressed genes between 41 and 48 DAT were obtained by filtering procedures of the t-test and fold change (FDR < 0.05 and FC > 3) and were used to generate the heat map. Based on similarity of expression patterns, we selected 357 upregulated and 333 downregulated genes. (b) Distribution of raw signal intensities of the upregulated and downregulated genes at 34, 41, 48 and 55 DAT. Click here for file

Additional file 12 **Differentially expressed genes from 41 DAT to 48 DAT**. Click here for file

Additional file 13 **Expression profile of miR399 and its target, *OsPHO2***. (a) Changes in expression of 11 miR399 precursors in leaf from 20 to 76 DAT. Error bars show s.e.m. (n = 3). (b) Changes in expression of *OsPHO2 *(Os05g0557700) in root. Microarray analysis was performed at weekly interval from 27 to 55 DAT with 3 replicates. Error bars represent s.e.m. (n = 3). Click here for file

Additional file 14 **Occurrence of PHR1 binding motif (GNATATNC) among differentially expressed genes from 41 DAT to 48 DAT**. Click here for file

Additional file 15 **Analysis of upregulated genes in leaf and mature pollen to clarify high-level transient gene expression at 90 DAT**. (a) Distribution of the raw signal intensity of the upregulated genes in the flag leaf at 83 DAT (blue bar) and 90 DAT (red bar). (b) Distribution of the raw signal intensity of all genes (35,760 genes) in mature pollen based on two replicate microarray data. (c) Distribution of the raw signal intensity of the upregulated genes in mature pollen. These genes include a large number of pollen-specific genes such as pollen-allergen genes and showed little expression in leaf before anthesis, but extremely high expression in mature pollen. We therefore concluded that the high-level of transient expression of these genes observed in leaf at 90 DAT was caused by pollen contamination of the leaf samples. Click here for file

Additional file 16 **Differentially expressed genes between 83 and 97 DAT**. (a) Hierarchical cluster analysis of 1,492 differentially expressed genes between 83 and 97 DAT selected by filtering procedures of the t-test and fold change (FDR < 0.05 and FC > 3). Cluster analysis was performed on relative expression values of all samples from 62 to 125 DAT. 1, 62-83 DAT; 2, 97-125 DAT. Transcriptome change was observed at 90 DAT (indicated by an asterisk). Yellow, black and blue scales indicate high, intermediate and low expression, respectively. We selected 573 downregulated genes and 423 upregulated genes on the basis of similarity of expression. (b) Relative expression values of six NAC transcriptional factors, which are contained in the 423 upregulated genes. Error bars represent s.e.m. (n = 3). Click here for file

Additional file 17 **Description of samples from fertile and sterile plants**. Click here for file

Additional file 18 **Expression of photosynthesis related genes in fertile and sterile plants**. The expression profiles of photosynthesis related genes as described in KEGG database [59], namely, osa00196 (Photosynthesis-antenna proteins) and osa00195 (Photosynthesis) in fertile and sterile plants were examined. The relative expression value of each gene was used for profiling. WAH: week(s) after heading. Click here for file
