# Supplementary material for: Field transcriptome revealed critical developmental and physiological transitions involved in the expression of growth potential in japonica rice
Source: BMC Plant Biol. 2011 Jan 12;11:10. doi: 10.1186/1471-2229-11-10 (PMC3031230; doi:10.1186/1471-2229-11-10)
Supplement: Additional file 1 — Overview of organ/tissue sampling performed to establish a field transcriptome of rice. Plant organs/tissues were sampled at various stages of the developmental cycle. Vegetative organs such as leaf blade, leaf sheath, root and stem were sampled at three specific points corresponding to the vegetative, reproductive and ripening stages recorded as number of days after transplanting (DAT) at both daytime (12:00) and nighttime (24:00). Sampling for different stages of development of inflorescence and anther was based on the length of the inflorescence and the anther itself, respectively, whereas sampling for pistil and lemma/palea was based on the length of the inflorescence and the floret, respectively. Sampling for ovary, embryo and endosperm was based on the number of days after flowering (DAF). For continuous gene expression profiling, the uppermost leaf in the main stem was sampled at weekly intervals from 13 to 125 DAT. Approximately 50% of the rice plants observed in the field at 56 DAT were at panicle initiation stage. At 58 DAT, almost 90% of the plants were at the early stage of panicle development indicating a complete reproductive transition. Then by 90 DAT, all plants were either in the flowering stage or early stages of seed development corresponding to the ripening stage transition. [file 1471-2229-11-10-S1.PDF]

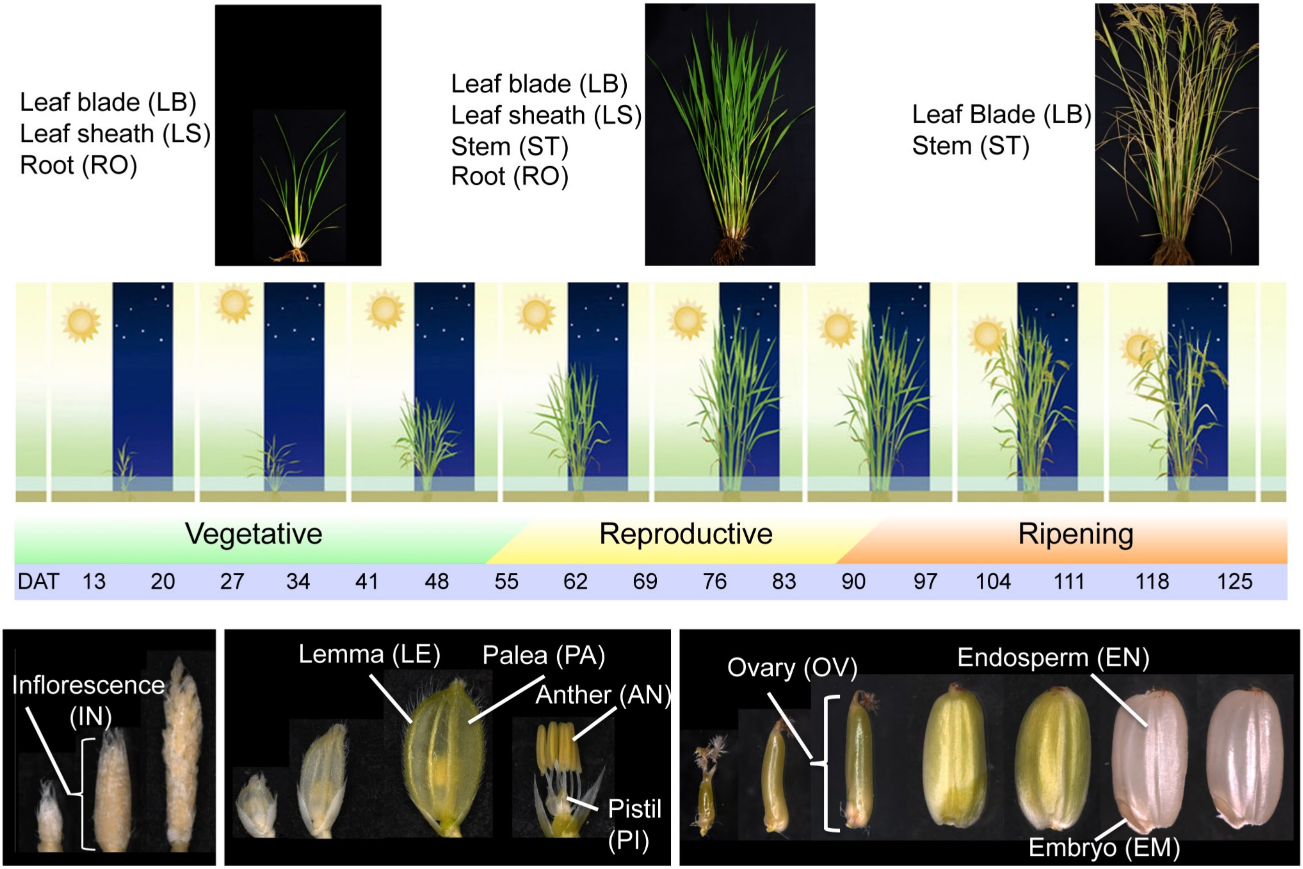

Additional file 1 - Overview of organ/tissue sampling performed to establish a field transcriptome of rice.

Plant organs/tissues were sampled at various stages of the developmental cycle. Vegetative organs such as leaf blade, leaf sheath, root and stem were sampled at three specific points corresponding to the vegetative, reproductive and ripening stages recorded as number of days after transplanting (DAT) at both daytime (12:00) and nighttime (24:00). Sampling for different stages of development of inflorescence and anther was based on the length of the inflorescence and the anther itself, respectively, whereas sampling for pistil and lemma/palea was based on the length of the inflorescence and the floret, respectively. Sampling for ovary, embryo and endosperm was based on the number of days after flowering (DAF). For continuous gene expression profiling, the uppermost leaf in the main stem was sampled at weekly intervals from 13 to 125 DAT. Approximately 50% of the rice plants observed in the field at 56 DAT were at panicle initiation stage. At 58 DAT, almost 90% of the plants were at the early stage of panicle development indicating a complete reproductive transition Then by 90 DAT, all plants were either in the flowering stage or early stages of seed development corresponding to the ripening stage transition.
