# Supplementary material for: Field transcriptome revealed critical developmental and physiological transitions involved in the expression of growth potential in japonica rice
Source: BMC Plant Biol. 2011 Jan 12;11:10. doi: 10.1186/1471-2229-11-10 (PMC3031230; doi:10.1186/1471-2229-11-10)
Supplement: Additional file 2 — Distribution of correlation coefficients calculated between biological replicates. The correlation coefficients obtained for three replicates in most samples were above 0.9, except for two stem samples suggesting high quality of gene expression data. [file 1471-2229-11-10-S2.PDF]

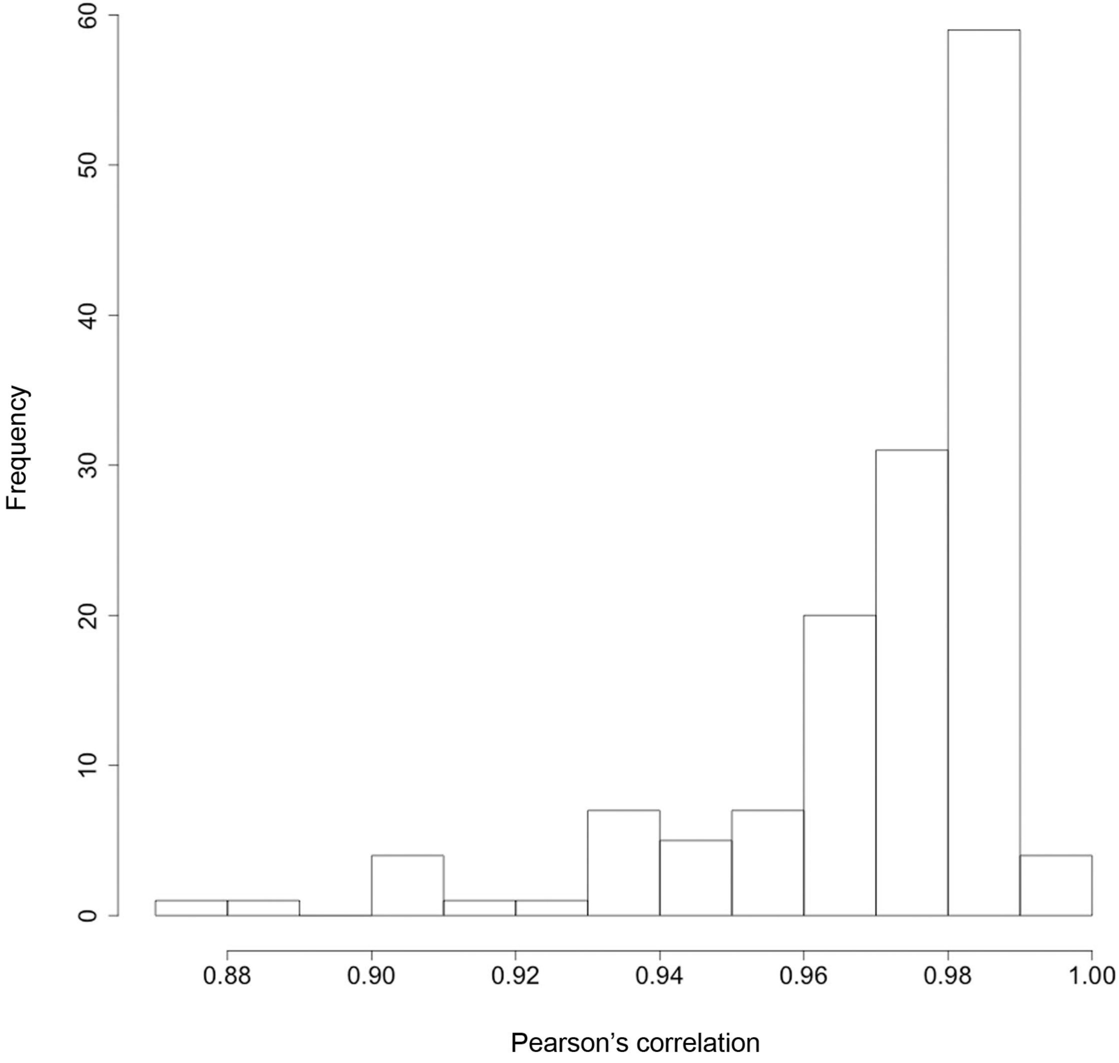

Additional file 2 - Distribution of correlation coefficients calculated between biological replicates.

The correlation coefficients obtained for three replicates in most samples were above 0.9, except for two stem samples suggesting high quality of gene expression data.
