# Supplementary material for: Field transcriptome revealed critical developmental and physiological transitions involved in the expression of growth potential in japonica rice
Source: BMC Plant Biol. 2011 Jan 12;11:10. doi: 10.1186/1471-2229-11-10 (PMC3031230; doi:10.1186/1471-2229-11-10)
Supplement: Additional file 3 — Hierarchical clustering of gene expression based on Gene Ontology categories. The average expression value for all genes under the same GO category was used to analyze relative gene expression level. All GO terms with relative expression level above 2.5 in at least one sample were extracted and hierarchical cluster analysis was performed using the R program. [file 1471-2229-11-10-S3.PDF]

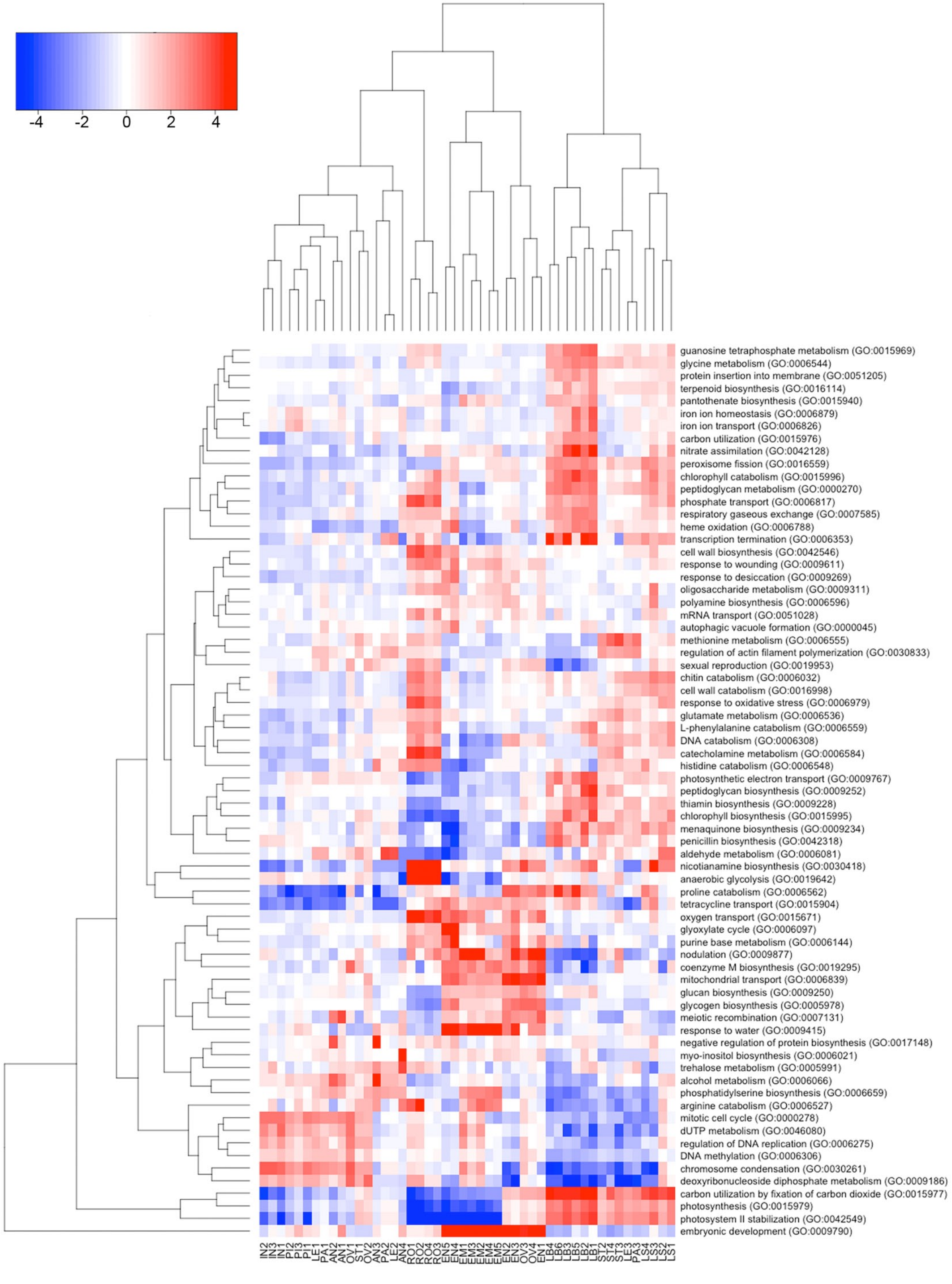

Additional file 3 - Hierarchical clustering of gene expression based on Gene Ontology categories.

The average expression value for all genes under the same GO category was used to analyze relative gene expression level. All GO terms with relative expression level above 2.5 in at least one sample were extracted and hierarchical cluster analysis was performed using the R program.
