# Supplementary material for: Field transcriptome revealed critical developmental and physiological transitions involved in the expression of growth potential in japonica rice
Source: BMC Plant Biol. 2011 Jan 12;11:10. doi: 10.1186/1471-2229-11-10 (PMC3031230; doi:10.1186/1471-2229-11-10)
Supplement: Additional file 5 — Expression profile of seed-specific transcription factors. Heat map of transcription factors highly expressed in developing seed, embryo and endosperm was constructed using the normalized signal intensity for each gene. High expression value is shown in red. [file 1471-2229-11-10-S5.PDF]

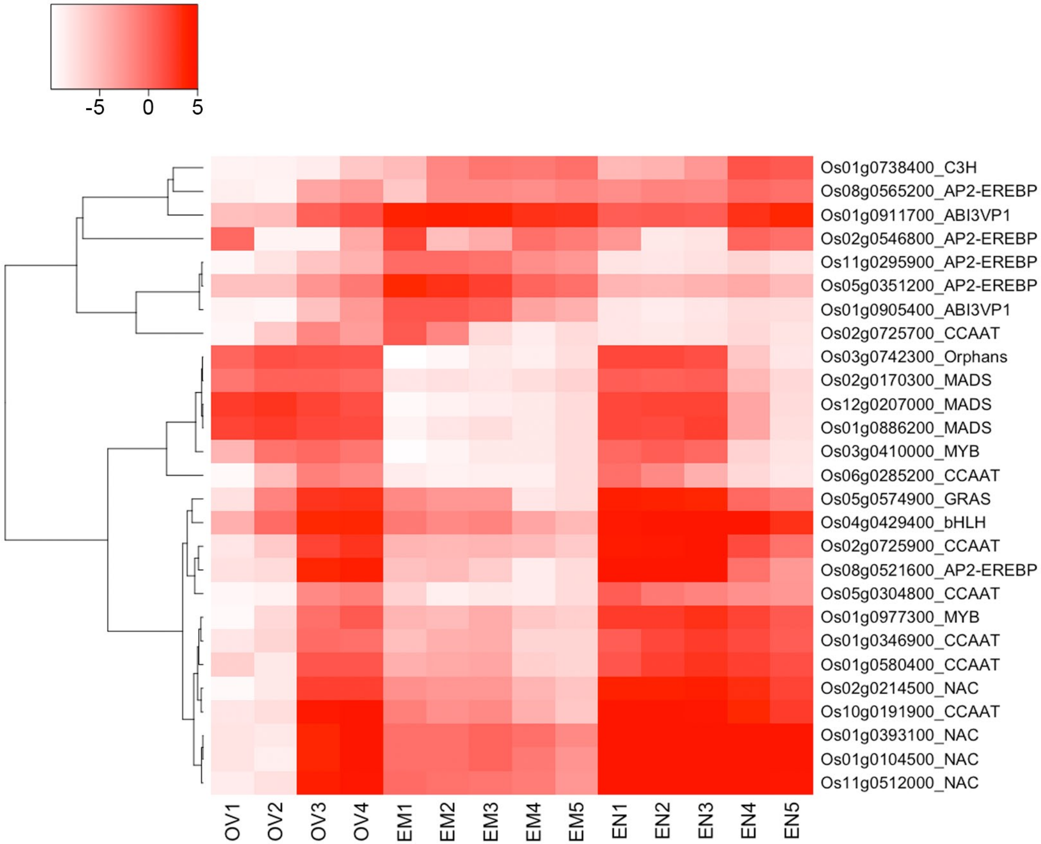

Additional file 5 - Expression profile of seed-specific transcription factors.

Heat map of transcription factors highly expressed in developing seed, embryo and endosperm was constructed using the normalized signal intensity for each gene. High expression value is shown in red.
