# Supplementary material for: Field transcriptome revealed critical developmental and physiological transitions involved in the expression of growth potential in japonica rice
Source: BMC Plant Biol. 2011 Jan 12;11:10. doi: 10.1186/1471-2229-11-10 (PMC3031230; doi:10.1186/1471-2229-11-10)
Supplement: Additional file 9 — Confirmation of the first major transcriptome change (a) Expression profile of leaf at weekly from 20 DAT to 62 DAT during the 2009 cultivation season. Microarray analysis was performed with two replicates in each point. The first transcriptome change and panicle differentiation was observed earlier as compared to the 2008 cultivation season. (b) Changes in expression level of Hd3a and RFT1. The red line represents RFT1 and the two blue lines represent the two probes for Hd3a. (c) Changes in expression level of five miR399 precursors. (d) PCA of the gene expression profile at 41, 48, and 62 DAT during 2008 cultivation season based on the uppermost leaf (1st leaf) in the main stem and the leaves below designed as the 2nd, 3rd, 4th, and 5th leaf from the uppermost leaf. Distinct clustering of the gene expression profiles at various positions supported the transcriptome change observed from 41 to 48 DAT. The number in each cluster represent the leaf position in the main stem. [file 1471-2229-11-10-S9.PDF]

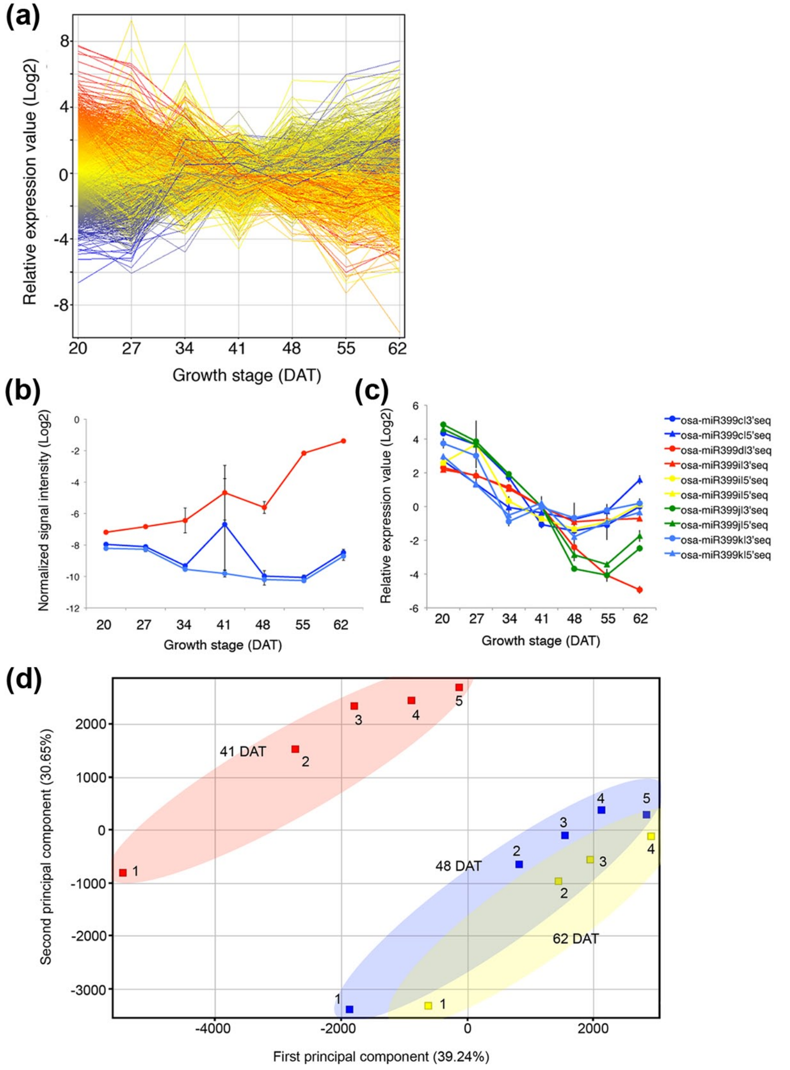

Additional file 9 – Confirmation of the first major transcriptome change

(a) Expression profile of leaf at weekly from 20 DAT to 62 DAT during the 2009 cultivation season. Microarray analysis was performed with two replicates in each point. The first transcriptome change and panicle differentiation was observed earlier as compared to the 2008 cultivation season. (b) Changes in expression level of *Hd3a* and *RFT1*. The red line represents *RFT1* and the two blue lines represent the two probes for *Hd3a*. (c) Changes in expression level of five miR399 precursors. (d) PCA of the gene expression profile at 41, 48, and 62 DAT during 2008 cultivation season based on the uppermost leaf (1st leaf) in the main stem and the leaves below designed as the 2nd, 3rd, 4th, and 5th leaf from the uppermost leaf. Distinct clustering of the gene expression profiles at various positions supported the transcriptome change observed from 41 to 48 DAT. The number in each cluster represent the leaf position in the main stem.
