# Supplementary material for: Field transcriptome revealed critical developmental and physiological transitions involved in the expression of growth potential in japonica rice
Source: BMC Plant Biol. 2011 Jan 12;11:10. doi: 10.1186/1471-2229-11-10 (PMC3031230; doi:10.1186/1471-2229-11-10)
Supplement: Additional file 10 — Expression profile of miR169 and its target gene, OsHAP2. (a) Changes in expression of 16 miR169 precursors from 20 to 76 DAT. Error bars show s.e.m. (n = 3). (b) Changes in expression of 8 OsHAP2 genes from 20 to 76 DAT. HAP genes without the miR169 target sites (OsHAP2A and OsHAP2B) did not show change in expression. Error bars represent s.e.m. (n = 3). The corresponding RAP-DB loci are as follows: OsHAP2A, Os08g0196700; OsHAP2B, Os12g0613000; OsHAP2C, Os03g0174900; OsHAP2D, Os03g0696300; OsHAP2E, Os03g0411100; OsHAP2F, Os12g0618600; OsHAP2G, Os07g0608200; OsHAP2H, Os03g0647600. [file 1471-2229-11-10-S10.PDF]

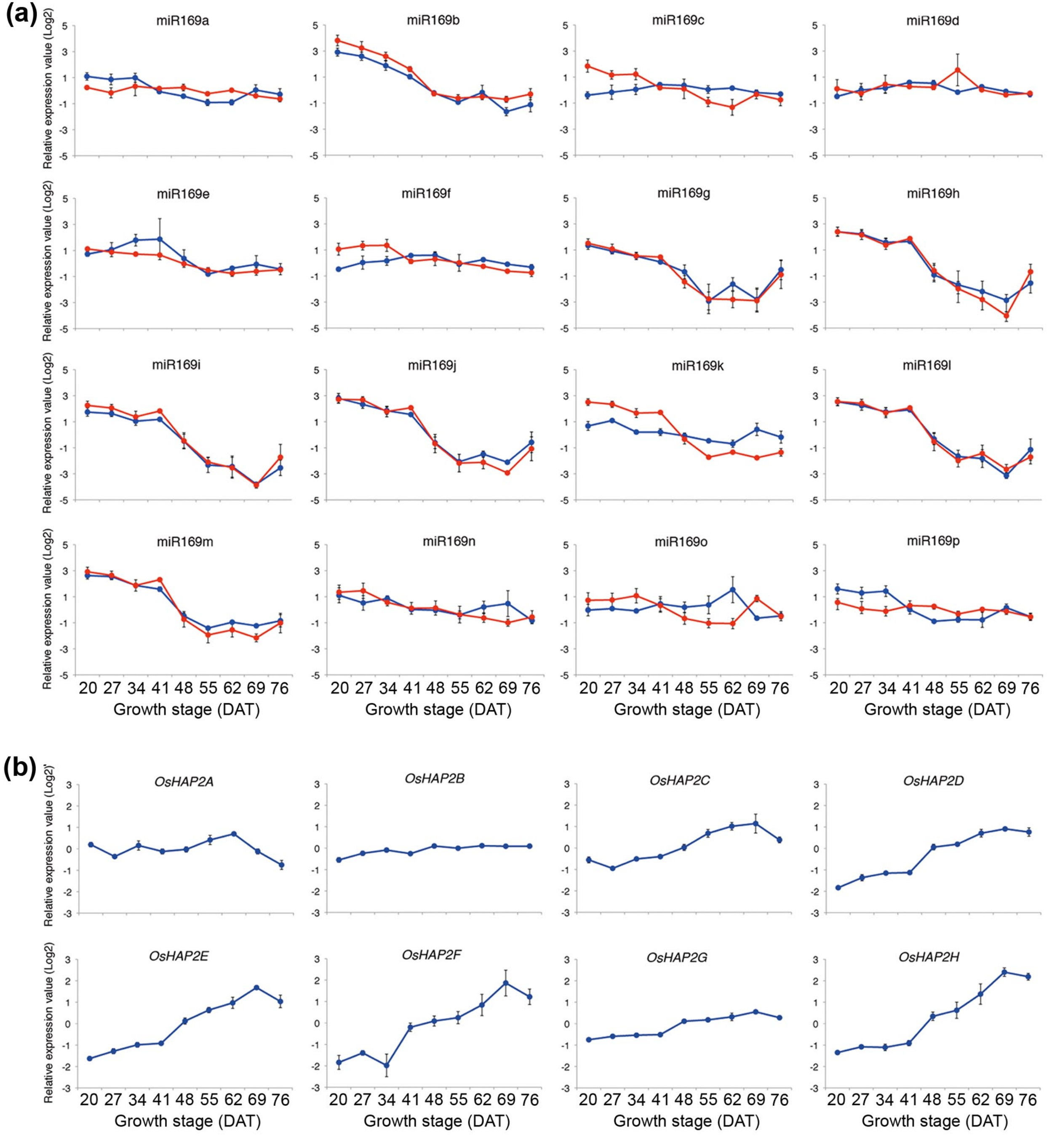

Additional file 10 - Expression profile of miR169 and its target gene, *OsHAP2*.

(a) Changes in expression of 16 miR169 precursors from 20 to 76 DAT. Error bars show s.e.m. (n=3). (b) Changes in expression of 8 *OsHAP2* genes from 20 to 76 DAT. HAP genes without the miR169 target sites (*OsHAP2A* and *OsHAP2B*) did not show change in expression. Error bars represent s.e.m. (n=3). The corresponding RAP-DB loci are as follows: *OsHAP2A*, Os08g0196700; *OsHAP2B*, Os12g0613000; *OsHAP2C*, Os03g0174900; *OsHAP2D*, Os03g0696300; *OsHAP2E*, Os03g0411100; *OsHAP2F*, Os12g0618600; *OsHAP2G*, Os07g0608200; *OsHAP2H*, Os03g0647600.
