# Supplementary material for: Field transcriptome revealed critical developmental and physiological transitions involved in the expression of growth potential in japonica rice
Source: BMC Plant Biol. 2011 Jan 12;11:10. doi: 10.1186/1471-2229-11-10 (PMC3031230; doi:10.1186/1471-2229-11-10)
Supplement: Additional file 11 — Differentially expressed genes between 41 and 48 DAT. (a) Hierarchical cluster analysis was performed on relative expression values of all samples from 20 to 76 DAT. A total of 1,316 differentially expressed genes between 41 and 48 DAT were obtained by filtering procedures of the t-test and fold change (FDR < 0.05 and FC > 3) and were used to generate the heat map. Based on similarity of expression patterns, we selected 357 upregulated and 333 downregulated genes. (b) Distribution of raw signal intensities of the upregulated and downregulated genes at 34, 41, 48 and 55 DAT. [file 1471-2229-11-10-S11.PDF]

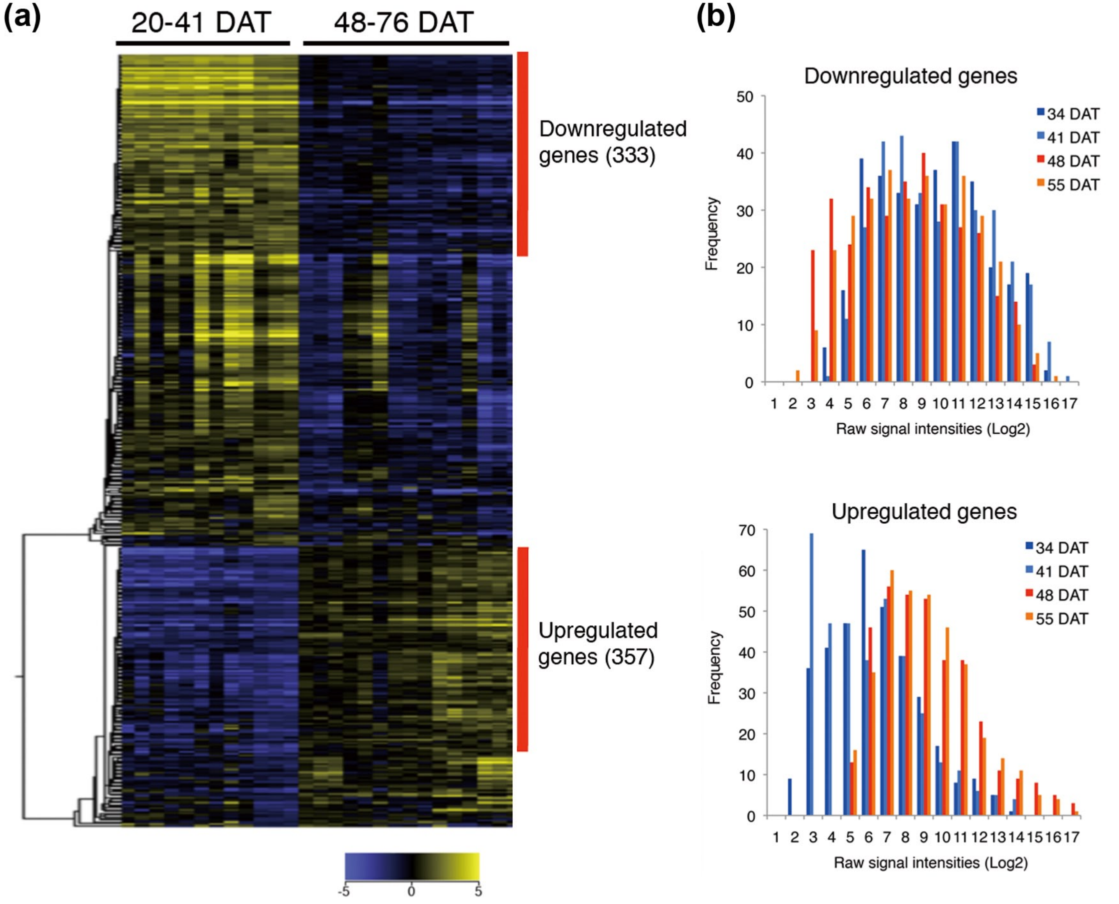

Additional file 11 - Differentially expressed genes between 41 and 48 DAT.

(a) Hierarchical cluster analysis was performed on relative expression values of all samples from 20 to 76 DAT. A total of 1,316 differentially expressed genes between 41 and 48 DAT were obtained by filtering procedures of the t-test and fold change (FDR<0.05 and FC>3) and were used to generate the heat map. Based on similarity of expression patterns, we selected 357 upregulated and 333 downregulated genes. (b) Distribution of raw signal intensities of the upregulated and downregulated genes at 34, 41, 48 and 55 DAT.
