# Supplementary material for: Field transcriptome revealed critical developmental and physiological transitions involved in the expression of growth potential in japonica rice
Source: BMC Plant Biol. 2011 Jan 12;11:10. doi: 10.1186/1471-2229-11-10 (PMC3031230; doi:10.1186/1471-2229-11-10)
Supplement: Additional file 13 — Expression profile of miR399 and its target, OsPHO2. (a) Changes in expression of 11 miR399 precursors in leaf from 20 to 76 DAT. Error bars show s.e.m. (n = 3). (b) Changes in expression of OsPHO2 (Os05g0557700) in root. Microarray analysis was performed at weekly interval from 27 to 55 DAT with 3 replicates. Error bars represent s.e.m. (n = 3). [file 1471-2229-11-10-S13.PDF]

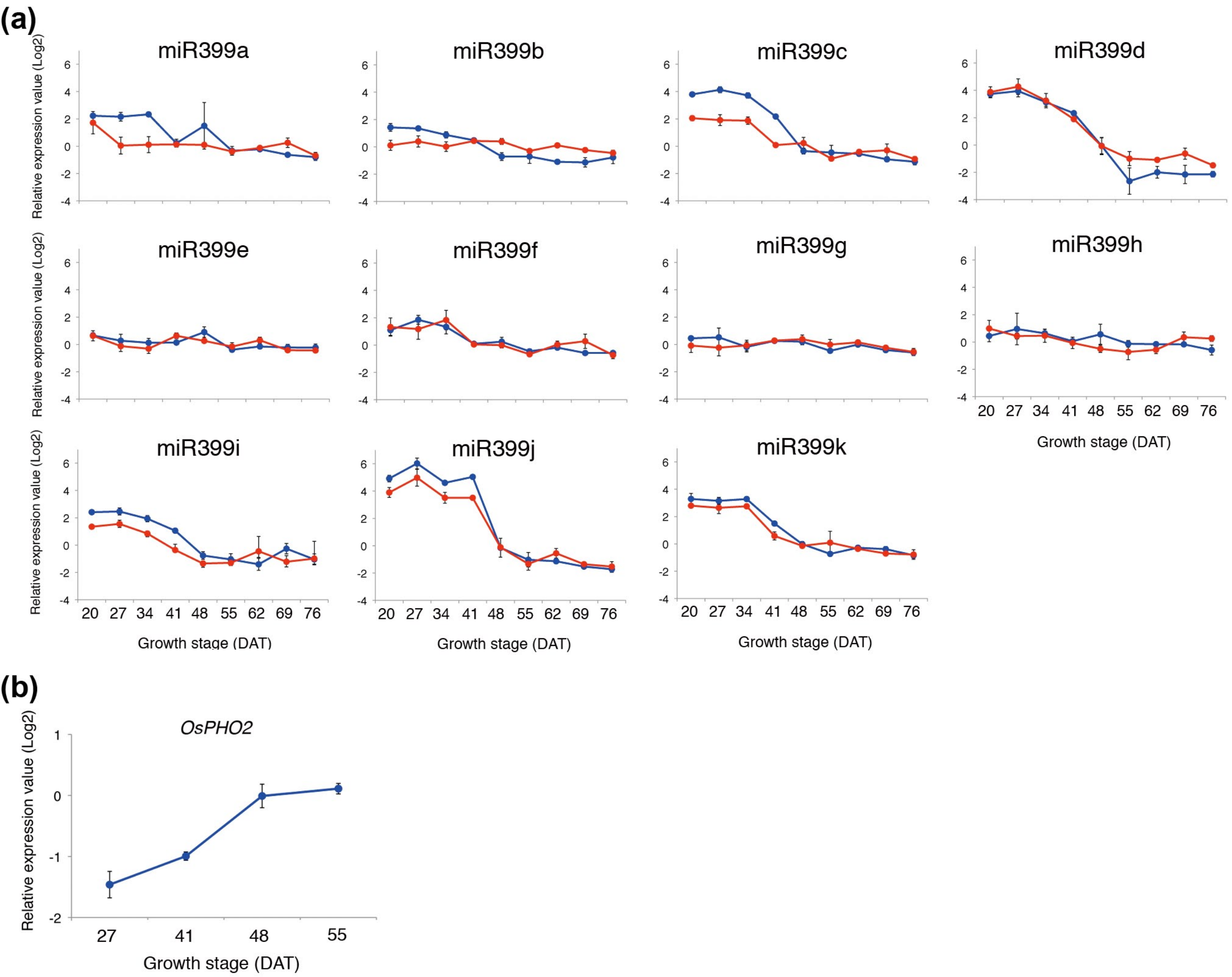

Additional file 13 - Expression profile of miR399 and its target, *OsPHO2*.

(a) Changes in expression of 11 miR399 precursors in leaf from 20 to 76 DAT. Error bars show s.e.m. (n=3). (b) Changes in expression of *OsPHO2* (Os05g0557700) in root. Microarray analysis was performed at weekly interval from 27 to 55 DAT with 3 replicates. Error bars represent s.e.m. (n=3).
