# Supplementary material for: Field transcriptome revealed critical developmental and physiological transitions involved in the expression of growth potential in japonica rice
Source: BMC Plant Biol. 2011 Jan 12;11:10. doi: 10.1186/1471-2229-11-10 (PMC3031230; doi:10.1186/1471-2229-11-10)
Supplement: Additional file 15 — Analysis of upregulated genes in leaf and mature pollen to clarify high-level transient gene expression at 90 DAT. (a) Distribution of the raw signal intensity of the upregulated genes in the flag leaf at 83 DAT (blue bar) and 90 DAT (red bar). (b) Distribution of the raw signal intensity of all genes (35,760 genes) in mature pollen based on two replicate microarray data. (c) Distribution of the raw signal intensity of the upregulated genes in mature pollen. These genes include a large number of pollen-specific genes such as pollen-allergen genes and showed little expression in leaf before anthesis, but extremely high expression in mature pollen. We therefore concluded that the high-level of transient expression of these genes observed in leaf at 90 DAT was caused by pollen contamination of the leaf samples. [file 1471-2229-11-10-S15.PDF]

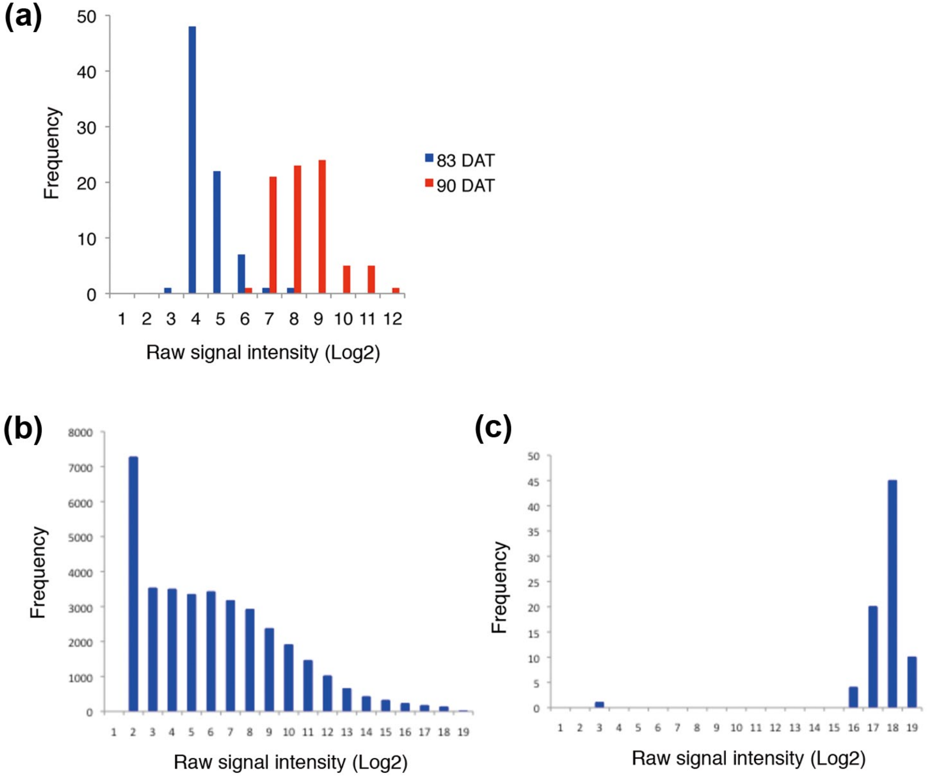

Additional file 15 - Analysis of upregulated genes in leaf and mature pollen to clarify high-level transient gene expression at 90 DAT.

(a) Distribution of the raw signal intensity of the upregulated genes in the flag leaf at 83 DAT (blue bar) and 90 DAT (red bar). (b) Distribution of the raw signal intensity of all genes (35,760 genes) in mature pollen based on two replicate microarray data. (c) Distribution of the raw signal intensity of the upregulated genes in mature pollen. These genes include a large number of pollen-specific genes such as pollen-allergen genes and showed little expression in leaf before anthesis, but extremely high expression in mature pollen. We therefore concluded that the high-level of transient expression of these genes observed in leaf at 90 DAT was caused by pollen contamination of the leaf samples.
