# Supplementary material for: Field transcriptome revealed critical developmental and physiological transitions involved in the expression of growth potential in japonica rice
Source: BMC Plant Biol. 2011 Jan 12;11:10. doi: 10.1186/1471-2229-11-10 (PMC3031230; doi:10.1186/1471-2229-11-10)
Supplement: Additional file 16 — Differentially expressed genes between 83 and 97 DAT. (a) Hierarchical cluster analysis of 1,492 differentially expressed genes between 83 and 97 DAT selected by filtering procedures of the t-test and fold change (FDR < 0.05 and FC > 3). Cluster analysis was performed on relative expression values of all samples from 62 to 125 DAT. 1, 62-83 DAT; 2, 97-125 DAT. Transcriptome change was observed at 90 DAT (indicated by an asterisk). Yellow, black and blue scales indicate high, intermediate and low expression, respectively. We selected 573 downregulated genes and 423 upregulated genes on the basis of similarity of expression. (b) Relative expression values of six NAC transcriptional factors, which are contained in the 423 upregulated genes. Error bars represent s.e.m. (n = 3). [file 1471-2229-11-10-S16.PDF]

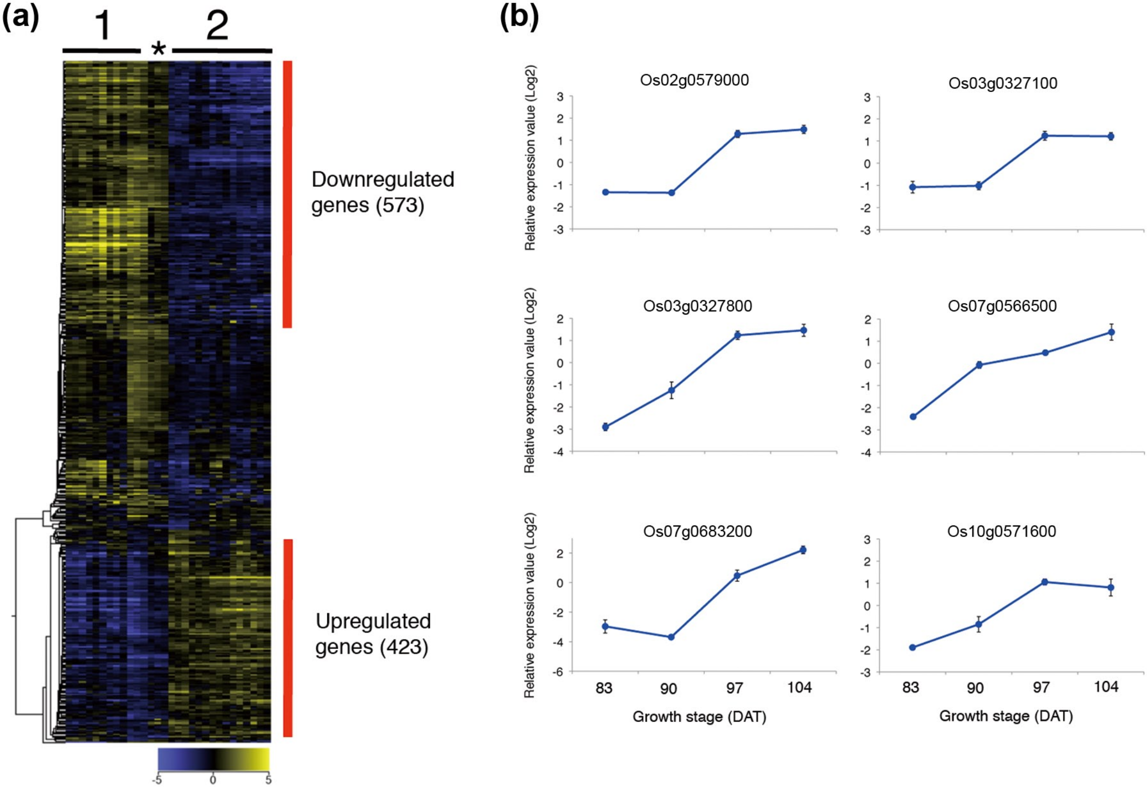

Additional file 16 - Differentially expressed genes between 83 and 97 DAT.

(a) Hierarchical cluster analysis of 1,492 differentially expressed genes between 83 and 97 DAT selected by filtering procedures of the t-test and fold change (FDR<0.05 and FC>3). Cluster analysis was performed on relative expression values of all samples from 62 to 125 DAT. 1, 62-83 DAT; 2, 97-125 DAT. Transcriptome change was observed at 90 DAT (indicated by an asterisk). Yellow, black and blue scales indicate high, intermediate and low expression, respectively. We selected 573 downregulated genes and 423 upregulated genes on the basis of similarity of expression. (b) Relative expression values of six NAC transcriptional factors, which are contained in the 423 upregulated genes. Error bars represent s.e.m. (n=3).
