# Supplementary material for: Field transcriptome revealed critical developmental and physiological transitions involved in the expression of growth potential in japonica rice
Source: BMC Plant Biol. 2011 Jan 12;11:10. doi: 10.1186/1471-2229-11-10 (PMC3031230; doi:10.1186/1471-2229-11-10)
Supplement: Additional file 18 — Expression of photosynthesis related genes in fertile and sterile plants. The expression profiles of photosynthesis related genes as described in KEGG database [59], namely, osa00196 (Photosynthesis-antenna proteins) and osa00195 (Photosynthesis) in fertile and sterile plants were examined. The relative expression value of each gene was used for profiling. WAH: week(s) after heading. [file 1471-2229-11-10-S18.PDF]

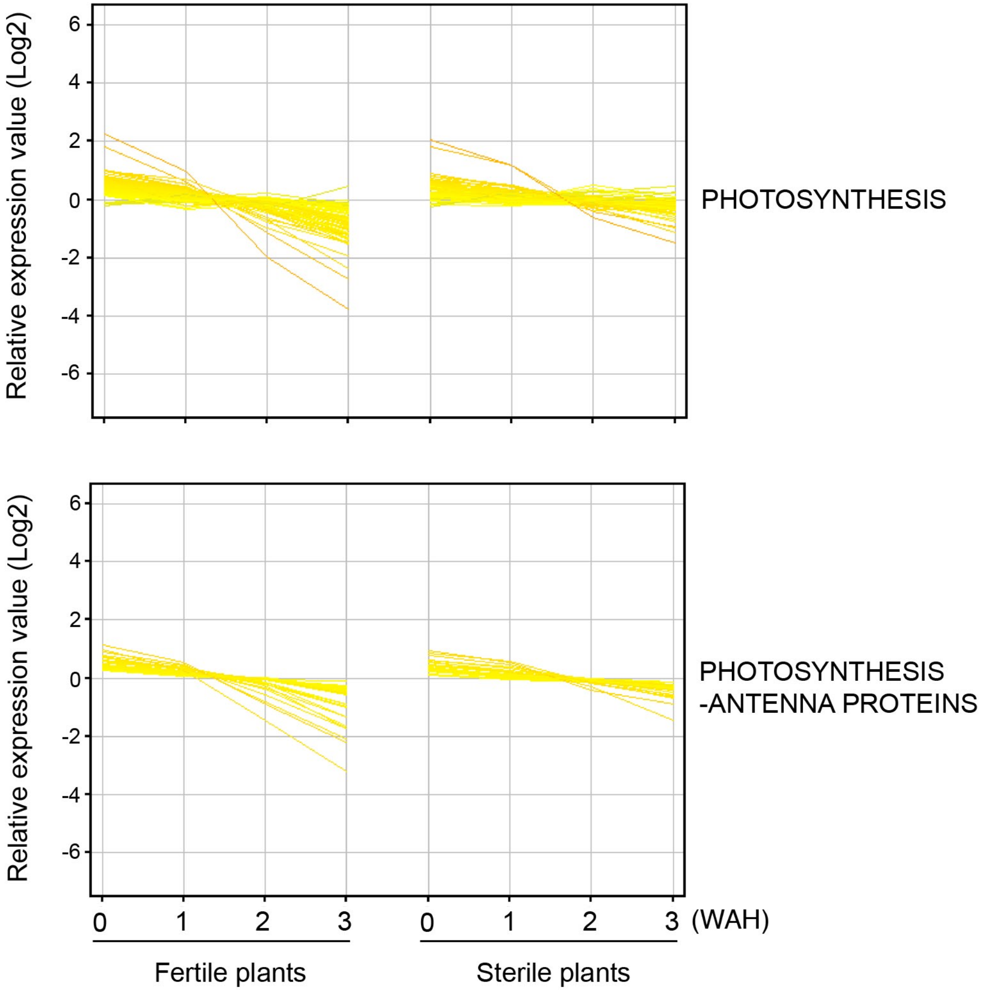

Additional file 18 - Expression of photosynthesis related genes in fertile and sterile plants. The expression profiles of photosynthesis related genes as described in KEGG database [59], namely, osa00196 (Photosynthesis-antenna proteins) and osa00195 (Photosynthesis) in fertile and sterile plants were examined. The relative expression value of each gene was used for profiling. WAH: week(s) after heading.
